# ScribbleDom: using scribble-annotated histology images to identify domains in spatial transcriptomics data

**DOI:** 10.1093/bioinformatics/btad594

**Published:** 2023-09-26

**Authors:** Mohammad Nuwaisir Rahman, Abdullah Al Noman, Abir Mohammad Turza, Mohammed Abid Abrar, Md Abul Hassan Samee, M Saifur Rahman

**Affiliations:** Department of Computer Science and Engineering, Bangladesh University of Engineering and Technology, Dhaka 1000, Bangladesh; Department of Computer Science and Engineering, Bangladesh University of Engineering and Technology, Dhaka 1000, Bangladesh; Department of Computer Science and Engineering, Bangladesh University of Engineering and Technology, Dhaka 1000, Bangladesh; Department of Computer Science and Engineering, Brac University, Dhaka 1212, Bangladesh; Department of Integrative Physiology, Baylor College of Medicine, Houston, TX 77030, United States; Department of Computer Science and Engineering, Bangladesh University of Engineering and Technology, Dhaka 1000, Bangladesh

## Abstract

**Motivation:**

Spatial domain identification is a very important problem in the field of spatial transcriptomics. The state-of-the-art solutions to this problem focus on unsupervised methods, as there is lack of data for a supervised learning formulation. The results obtained from these methods highlight significant opportunities for improvement.

**Results:**

In this article, we propose a potential avenue for enhancement through the development of a semi-supervised convolutional neural network based approach. Named “ScribbleDom”, our method leverages human expert’s input as a form of semi-supervision, thereby seamlessly combines the cognitive abilities of human experts with the computational power of machines. ScribbleDom incorporates a loss function that integrates two crucial components: similarity in gene expression profiles and adherence to the valuable input of a human annotator through scribbles on histology images, providing prior knowledge about spot labels. The spatial continuity of the tissue domains is taken into account by extracting information on the spot microenvironment through convolution filters of varying sizes, in the form of “Inception” blocks. By leveraging this semi-supervised approach, ScribbleDom significantly improves the quality of spatial domains, yielding superior results both quantitatively and qualitatively. Our experiments on several benchmark datasets demonstrate the clear edge of ScribbleDom over state-of-the-art methods—between 1.82% to 169.38% improvements in adjusted Rand index for 9 of the 12 human dorsolateral prefrontal cortex samples, and 15.54% improvement in the melanoma cancer dataset. Notably, when the expert input is absent, ScribbleDom can still operate, in a fully unsupervised manner like the state-of-the-art methods, and produces results that remain competitive.

**Availability and implementation:**

Source code is available at Github (https://github.com/1alnoman/ScribbleDom) and Zenodo (https://zenodo.org/badge/latestdoi/681572669).

## 1 Introduction

Spatial transcriptomics (ST), an emerging technology to profile gene expression at the spatial resolution, can reveal new insights into molecular aspects of tissue architecture (Longo *et al.* 2021, Rao *et al.* 2021, Moses and Pachter 2022, Palla *et al.* 2022). One such aspect is transcriptional homogeneity in tissue regions. Since ST data captures gene expression at spatially resolved spots, clustering the spots based on their transcriptomic profiles can demarcate transcriptionally homogeneous tissue regions (Maynard *et al.* 2021). The goal here is to identify spatial domains in the ST data, similar to how image segmentation algorithms demarcate objects in images. Notably, most image segmentation algorithms are “supervised”, i.e., they require training examples with segments demarcated *a priori*. However, such training examples are scarce in the realm of ST, requiring the spatial domain identification algorithms to be unsupervised. Some of the methods that try to solve this problem are Giotto (Dries *et al.* 2021), stLearn (Pham *et al.* 2020), SpaGCN (Hu *et al.* 2021), BayesSpace (Zhao *et al.* 2021), SC-MEB (Yang *et al.* 2022), etc., of which, SC-MEB, BayesSpace, and SpaGCN are state-of-the-art methods that demonstrated quantitatively better performance than others (Table 1). BayesSpace implements a fully Bayesian statistical method that uses the information from spatial neighborhoods for resolution enhancement of spatial transcriptomic data. SC-MEB, on the other hand, implements an empirical Bayes approach for spatial clustering analysis using a hidden Markov random field. SpaGCN applies a graph convolutional network approach to integrate gene expression profile data, spatial location as well as histology. These approaches for clustering analysis have demonstrated concordance with the available handful of “gold-standard” datasets.

**Table 1.** Comparison of ScribbleDom and AutoScribbleDom with state-of-the-art spatial and non-spatial clustering methods based on ARI.^a^

Sample	ScribbleDom	AutoScribbleDom	SC-MEB	BayesSpace	SpaGCN	Giotto	GMM	Louvain
151507	**0.53**	0.34	0.42	0.33	0.49	0.33	0.40	0.32
151508	0.37	**0.44**	**0.44**	0.36	0.43	0.34	0.33	0.25
151509	**0.65**	0.42	0.52	0.44	0.44	0.35	0.29	0.30
151510	0.46	**0.55**	0.39	0.43	0.45	0.33	0.31	0.28
151669	**0.66**	0.32	0.32	0.41	0.26	0.25	0.22	0.20
151670	**0.70**	0.33	0.43	0.43	0.37	0.21	0.19	0.26
151671	**0.71**	0.60	0.42	0.38	0.52	0.40	0.23	0.36
151672	0.74	0.63	0.44	**0.77**	0.57	0.38	0.14	0.27
151673	0.50	0.52	0.49	**0.55**	0.53	0.37	0.29	0.29
151674	**0.54**	0.25	0.43	0.33	0.39	0.29	0.29	0.33
151675	**0.52**	0.39	0.31	0.41	0.46	0.32	0.24	0.24
151676	**0.51**	0.38	0.39	0.32	0.35	0.26	0.26	0.25

a The ARI values of SpaGCN have been collected from the respective paper (Hu *et al.* 2021). The ARI values of the remaining state-of-the-art methods were collected from SC-MEB (Yang *et al.* 2022) paper. The best ARI value for each sample has been marked in bold-face.

To enhance the biological relevance of the spatial domains in ST data, we propose a “scribble-supervised” spatial domain identification method, “ScribbleDom”. This semi-supervised approach shares similarities with semi-supervised image segmentation methods and is unattainable by repurposing the existing state-of-the-art methods. An investigation of convolutional neural networks (CNNs) for unsupervised image segmentation was done by Kim *et al.* (2020). They proposed a novel unsupervised image segmentation end-to-end network that includes normalization and an argmax function for differentiable clustering. They also have the option to include prior knowledge about a subset of pixels, obtained through the drawing of scribbles over the input image, which is widely utilized in academic research and commercial applications and is widely regarded as one of the most user-friendly methods of interaction. Using scribbles as annotations, ScribbleSup (Lin *et al.* 2016) demonstrated competitive object semantic segmentation results on the PASCAL VOC dataset. To provide pixel-wise labeling, Xu *et al.* (2015) presented a unified strategy that integrates several forms of weak supervision—image-level tags, bounding boxes, and partial labels. CNNs are widely used for image classification, segmentation, and object detection. Xie *et al.* (2015) proposed deep embedded clustering (DEC), a method that uses deep neural networks to simultaneously learn feature representations and cluster allocations. The use of a CNN-based approach with three-dimensional filters on hand and brain MRI is presented by Kayalibay *et al.* (2017). Wang *et al.* (2018) proposed image-specific fine-tuning to adapt a CNN model to a given test image, which can be either unsupervised (with no extra user interactions) or supervised (with additional scribbles).

ScribbleDom takes inspiration from Kim *et al.*’s (2020) semi-supervised formulation of the image segmentation problem. Subsequently, we have significantly customized the model architecture as well as the loss function to cater to the problem of identifying spatial domains in ST data. ST data contains tissue domains with a diverse range of narrowness. To effectively handle the variability in the narrowness of spatial domains in ST data, ScribbleDom has incorporated Inception blocks (Szegedy *et al.* 2015) instead of vanilla convolutions. Inception blocks allow the model to process ST data at various viewpoints by employing convolution filters of different sizes. Thus the model is able to capture fine-grained details in narrow spatial domains while also considering broader spatial contexts. By combining the output from the different-sized filters, ScribbleDom effectively integrates information from various levels of granularity, allowing for a comprehensive understanding of the spatial organization within the ST data. ScribbleDom is broadly applicable to technologies that do not generate matching pairs of histology image and RNA data (see Section 4).

**Figure 1.**
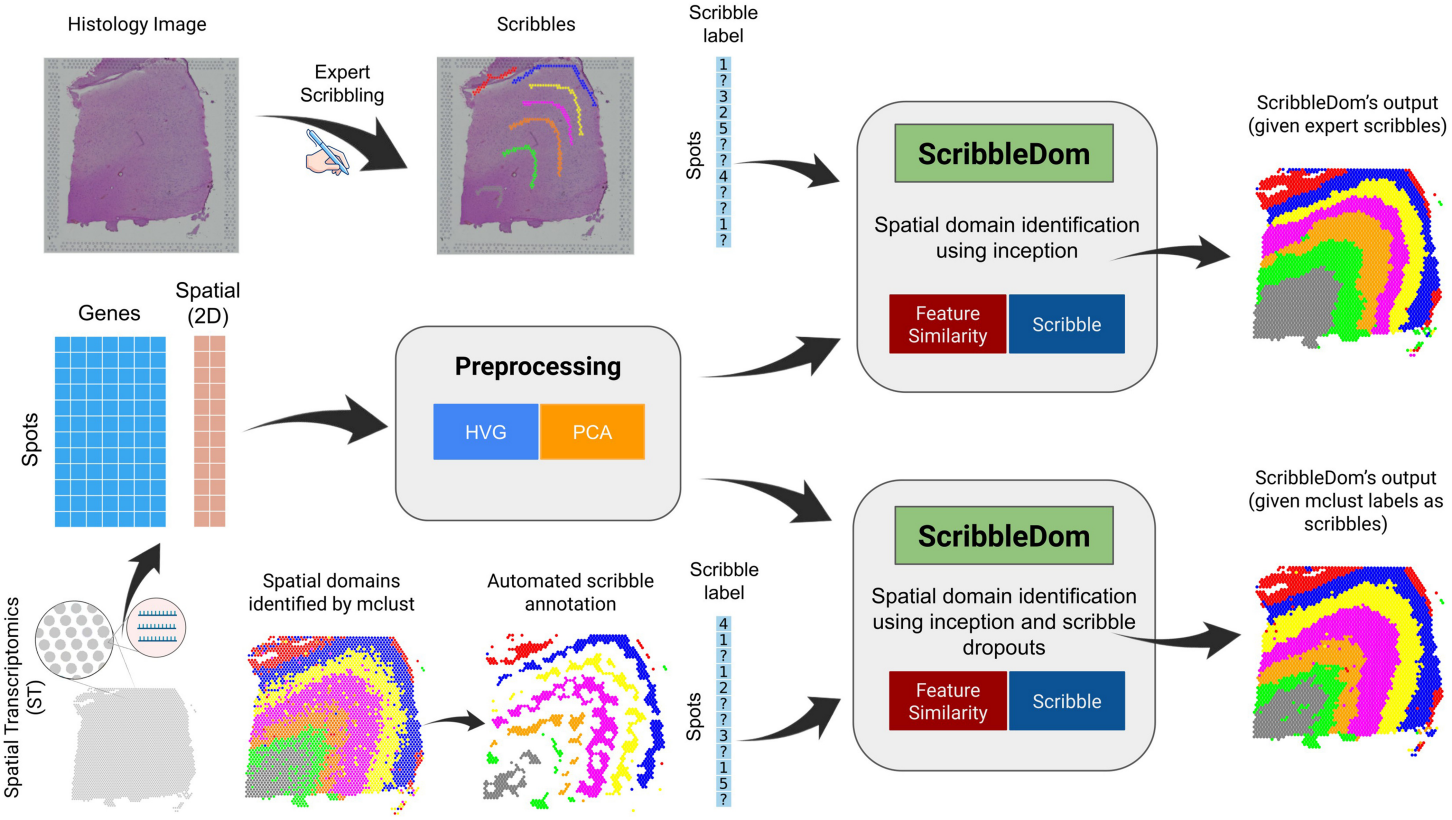
ScribbleDom overview. ScribbleDom can work through two different pipelines, when the human annotator scribbles over the histology image (upper part) and when the output of a non-spatial clustering algorithm (e.g. mclust) is used as prior knowledge (lower part). ScribbleDom receives the prior knowledge about the spots, the preprocessed transcriptomics data, and the spatial information about each spot as its input. Data are preprocessed by taking highly variable genes (HVG) and then performing principal component analysis (PCA) on these HVGs. ScribbleDom identifies domains in the ST data using Inception by minimizing a loss function having two components—feature similarity loss and scribble loss. The results produced by ScribbleDom show significant improvement compared to state-of-the-art models.

ScribbleDom makes use of the scribble information provided by a human expert. However, in the absence of scribbles from a human annotator, ScribbleDom can still work to produce competitive results by obtaining initial labeling from another clustering algorithm to run in a completely unsupervised fashion. An overview of the workflow in both modes is shown in Fig. 1. Many of the state-of-the-art methods (e.g. BayesSpace, SC-MEB, SpaGCN, etc.) also incorporate this approach of initializing cluster labels by another clustering algorithm. BayesSpace and SC-MEB use mclust (Scrucca *et al.* 2016) and SpaGCN uses Louvain’s method (De Meo *et al.* 2011) for initialization. Using the samples from the dataset of the human brain’s dorsolateral prefrontal cortex (DLPFC) region (Visium), melanoma (ST), and human breast cancer (Visium), we have demonstrated that ScribbleDom outperforms state-of-the-art models like BayesSpace, SpaGCN, and SC-MEB in the presence of expert’s scribble annotations. In case the scribble annotations are unavailable, ScribbleDom can still operate in fully unsupervised manner and its performance remains competitive with the state-of-the-art methods.

The key contributions of the paper can be summarized as follows.

- We have proposed ScribbleDom, a semi-supervised deep-learning-based approach to identify spatial domains in ST data. ScribbleDom allows for seamless combination of human cognitive ability with machines’ computational power for spatial domain identification in ST data using scribble-annotated histology images. This approach makes it possible to change the course of the spatial domain identification procedure through different types of scribbles.
- We have devised a scoring function (goodness score) to rank the clustering outcomes from different algorithms, or the same algorithm for different hyperparameter settings. The scoring function takes into consideration gene expression similarity and spatial continuity, and does not require manual annotation for its calculation, unlike metrics such as adjusted Rand index (ARI), Dice score, etc. We have applied this goodness measure in ScribbleDom to choose the optimal hyperparameter settings in a sample-specific manner.
- We have analyzed several samples using ScribbleDom and have successfully identified the spatial domains therein. These samples span from tissues that mostly have layers as their spatial domains (DLPFC samples) to tissues with diversity of different spatial structures, such as tumorous cell clusters embedded in healthy tissues (melanoma and human breast cancer datasets). The output of ScribbleDom is generally superior to that of state-of-the-art methods, both qualitatively and quantitatively

## 2 Materials and methods

### 2.1 Dataset

In this study, we analyzed three different datasets. Firstly, we utilized a dataset consisting of 12 human brain samples extracted from the DLPFC region. These samples were measured using the 10x Genomics (https://www.10xgenomics.com/) Visium platform. Each sample, when observed in a perpendicular tissue segment, encompasses all six layers of the cortex as well as the adjacent white matter. Maynard *et al.* (2021) annotated the DLPFC layers by considering cytoarchitecture and selected gene markers. The full dataset is publicly available through the spatialLIBD (Maynard *et al.* 2021) Bioconductor package. This is a gold-standard dataset in the literature as it contains manual annotation for each of the samples. Each of the samples has approximately 4000 spots on average and spots that were manually annotated belong to one of six DLPFC layers or white matter. As the samples were obtained via Visium technology, each of the spots has six neighboring spots, and these six spots correspond to six vertices of a hexagon. Gene expression profile was also available. Each of the spots approximately has 33 000 gene expression values. The spatial information is available in the form of coordinates of each of the spots. One of the challenges of this dataset is to recapitulate the number of layers and the narrowness of each of the layers. While we have analyzed all the samples, we have described our results only for sample 151673 in the main text of the paper. This is because state-of-the-art methods like BayesSpace and SpaGCN showcase their result using this sample. Also, the output for this sample provides better visual quality than the other ones with respect to the narrowness of layers.

In the second dataset, we focus on analyzing the melanoma cancer samples that were processed using the ST platform (Thrane *et al.* 2018). Specifically, we analyzed the second replicate from biopsy 1, because it contains manual annotation where the tissue regions are dispersed across spatially diverse areas. Biopsy 1 consisted of a total of 293 spots covered by tissue.

For the third dataset, we utilized publicly available data from the 10x Genomics (Zheng *et al.* 2017) website. This dataset included spatial transcriptomics data of human breast cancer obtained using the Visium platform. This is ductal carcinoma tissue where the tumors are spread over various regions. It comprises a total of 2518 spots. We obtained the manual annotation from Ni *et al.* (2022) where they classify each spot as tumor or non-tumor.

### 2.2 Data preprocessing

In spatial transcriptomics, a transcript count matrix ($X=[x_{ij}]\in N^{n\times g}$) and spatial coordinates ($Z=[z_{ij}]\in R^{n\times 2}$) are generated for *n* different locations (spots) on a tissue slice, where *g* represents the number of genes. To preprocess the count matrix, we utilized the *spatialPreprocess* method from the BayesSpace R package. The data underwent log normalization, and the function was able to select *h* highly variable genes and extract *p* principal components (PCs). The processed count matrix ($X\prime =[x\prime_{ij}]\in R^{n\times p}$) is used for the spatial domain identification.

In our experiment, we chose 2000 highly variable genes for all datasets. The number of principal components was determined based on the BayesSpace approach, with the top 15 and 7 principal components selected for the human DLPFC and melanoma datasets, respectively. For the human breast cancer dataset, we analyzed the scree plot of the top 50 principal components and chose 15 principal components for analysis (see Supplementay data for the scree plot).

We analyzed datasets that were generated using different spatial transcriptomic technologies, namely ST and Visium. While these technologies have distinct spot distribution patterns, we need to map each spot to a cell of a data matrix so that conventional convolution filters can be applied. This mapping is trivial for ST data, as the spots are organized in square lattice. However, the Visium spots are arranged in a hexagonal lattice structure, with each spot having six neighboring spots. Therefore, we preprocessed the Visium data such that the six neighbors of a spot located at grid location (*i*, *j*) are spots positioned at (i+1,j), (i−1,j), (i,j+1), (i,j−1), (i−1,j−1), and (i+1,j+1).

### 2.3 Scribble generation

To facilitate the semi-supervised approach in ScribbleDom, scribbles were generated for the Visium and ST data. For Visium data, this process utilized the CLOUPE file provided by 10x Genomics, which contains essential information regarding histology and spot mapping for the tissue samples. Using Loupe browser 6.3.0, a tool developed by 10x Genomics, we annotated various Visium spots within the tissue. In the case of ST data, specifically for melanoma sample, we scribbled over the pixels of the corresponding histology image. The pixels that were scribbled were then mapped to the corresponding ST spot array. This process allowed us to associate the scribbles with specific spots in the ST data.

When human annotations (scribbles) are not available, scribbles are generated automatically based on an initial clustering generated by mclust, a non-spatial clustering algorithm. We call this pipeline *AutoScribbleDom*. As mclust is a non-spatial clustering algorithm, the initial clustering thus produced is expected to have inaccuracies in the spatial domains identified. Therefore, only the spots with labels identical to those of all its neighboring spots (six neighbors for Visium, four neighbors for ST) are considered as scribbles, while the labels from the remaining spots are cleared out. The rest of the pipeline is identical to ScribbleDom.

### 2.4 Model architecture

**Figure 2.**
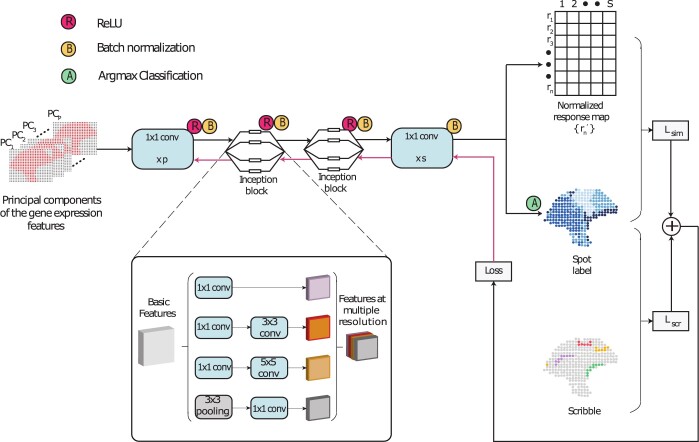
Schematic diagram of the model architecture of ScribbleDom, which consists of two 1 × 1 convolutions and two inception blocks. The inception blocks facilitate capturing features at different resolutions. Loss is calculated using cross entropy between the predicted cluster label and the scribble (for the scribbled spots) or the response map (for the spots without scribbles).

A high-level model architecture of ScribbleDom is shown in Fig. 2. First, the input tensor containing the principal components of each spot is fed into a 1 × 1 convolution layer with *p* channels. Then there are two inception blocks, which enable the model to extract features at multiple resolutions. After each of these layers, ReLU is used for non-linear activation, which is followed by batch normalization. Subsequently, the features undergo another 1 × 1 convolution layer and batch normalization, resulting in an *s*-dimensional normalized response map for each spot, where *s* is the number of spatial domains to be identified. Finally, each spot’s spatial domain label is obtained by taking the argmax on the response map. The loss function is calculated based on the response map, spot labels, and scribble labels (more details follow).

An inception block consists of parallel information paths consisting of 1 × 1, 3 × 3, 5 × 5 convolution filters, and a 3 × 3 pooling layer. Features extracted alongside these paths are finally concatenated (Fig. 2, blowout). The first inception block in our model extracts 256 features where, 160, 64, 16, and 16 features are extracted from the aforementioned paths, respectively. The next inception block extracts 96, 16, 8, and 8 features, respectively, resulting in a concatenation of 128 features. The filters of varied sizes enable the extraction of features at multiple resolutions, aligning with the notion that information should be processed and aggregated across scales. The smaller filters contribute to capturing fine-grained features, while the larger filters facilitate the inclusion of features from the neighboring spots. This amalgamation of features has proven to be advantageous in our experimental evaluations.

### 2.5 Loss function

As inputs to our model, we utilized the low-dimensional representation of the transcript count matrix ($X\prime \in R^{n\times p}$), the spatial location of the tissue slice spots ($Z\in R^{n\times 2}$), and the number of spatial domains to be identified (*s*). If an expert provided scribbles, then *s* was equal to the number of differently colored scribbles. In the case of AutoScribbleDom, we provided the value of *s* as an input to the non-spatial clustering algorithm, mclust. For each spot, we determine the optimal label from {1,…,s} by minimizing the following two-component loss function:


$$\alpha \cdot L_{\mathrm{sim}}\left({\left\{r_{i},c_{i},u_{i}\right\}}_{i=1}^{n}\right)+(1-\alpha )\cdot \sum_{j=1}^{s}L_{\mathrm{scr}}\left({\left\{r_{i},w_{i},u_{i}\right\}}_{i=1}^{n}\right)$$

where *α* is a hyperparameter. Here $r_{i}\in R^{s}$ is the output of a deep neural network that maps the gene expression data of the *i*-th spot to an *s*-dimensional vector. The loss function’s first component is:


$$L_{\mathrm{sim}}\left({\left\{r_{i},c_{i},u_{i}\right\}}_{i=1}^{n}\right)=\sum_{i=1}^{n}\sum_{j=1}^{s}-(1-u_{i})\delta \left(j,c_{i}\right)\ln\left(r_{i,j}\right)$$

where *δ* is the Kronecker delta function and $c_{i}=\underset{k}{\arg\max}r_{i,k}$ is the predicted label for this spot. *u_i_* = 1 if the *i*-th spot overlaps a scribble, *u_i_* = 0 otherwise. Intuitively, minimizing $L_{\mathrm{sim}}$ minimizes variance in the predicted values of *c_i_*. The second component is:


$$L_{\mathrm{scr}}\left({\left\{r_{i},w_{i},u_{i}\right\}}_{i=1}^{n}\right)=\sum_{i=1}^{n}\sum_{j=1}^{s}-u_{i}\delta \left(j,w_{i}\right)\ln\left(r_{i,j}\right)$$

where $w_{i}\in \{1,\ldots ,s\}$ is the scribble label of spot *i*. Thus, minimizing *L_scr_* imposes the constraint that *c_i_* matches scribble label of spot *i*.

### 2.6 Scribble dropout

In case of AutoScribbleDom, the initial spot labels from mclust that conforms with neighboring spot labels are considered as scribbles. Nevertheless there can still be some noise in the scribbles. To address this issue, during training, we randomly remove scribble annotations from some spots in each iteration, much like the concept of dropout in deep learning networks. This approach allows the model to learn similarity with other spots, even if some of the spots are incorrectly labeled by the automated scribbles. The dropout rate is controlled by a second hyperparameter called *β*.

### 2.7 Hyperparameter selection

ScribbleDom has only one hyperparameter, *α*, which can tune the relative contribution of similarity loss and the scribble loss to the overall loss function. AutoScribbleDom has a second hyperparameter, *β*, that controls the rate of scribble dropout. We performed a grid search in the hyperparameter space to find the optimal values. We searched *α* over 0.05 to 0.95 and *β* over 0.25 to 0.4, each with an interval of 0.05. The best values were determined by comparing the clustering outputs based on a goodness measure that is described below.

Let the cluster assignment for *n* spots generated by our model for a particular value of *α* be $C^{\alpha }=[C_{1}^{\alpha },C_{2}^{\alpha },\ldots ,\;C_{n}^{\alpha }]$. Here, $C_{i}^{\alpha }\in \{1,\ldots ,s\}$ is the cluster label for the *i*-th spot and *s* is the number of clusters. For different values *α*, this clustering output would naturally be different. To select the optimal *α*, we developed a scoring function to measure the goodness of each clustering output. This goodness measure consists of three components. The first component is the likelihood of the gene expression PCs of the spots given their cluster assignments, $p\left(y|C^{\alpha }\right)=\prod_{i=1}^{n}p\left(y_{i}|C_{i}^{\alpha }\right)$. Here *y_i_* is the gene expression PC vector at spot *i*. We assume that the PCs are Gaussian distributed within each cluster j∈{1,…,s} with mean $\mu_{j}=\mathrm{Average}\left(\left\{y_{i}|1\leq i\leq n,\;C_{i}^{\alpha }=j\right\}\right)$ and covariance matrix $\Sigma_{j}=\mathrm{Covariance}\left(\left\{y_{i}|1\leq i\leq n,\;C_{i}^{\alpha }=j\right\}\right)$. The intuition for this component is that if a spot is assigned to its correct cluster, it will have a higher likelihood compared to had it been assigned to a wrong cluster.

The second component is the likelihood of the cluster assignment itself, $p(C^{\alpha })=\prod_{i=1}^{n}p(C_{i}^{\alpha })$, where $p(C_{i}^{\alpha })$ is given by the Potts model (Wu 1982).


$$p(C_{i}^{\alpha })=\frac{1}{Z_{i}}\text{exp}\;\left(\frac{2\gamma }{\left|\langle ij\rangle \right|}\sum_{\langle ij\rangle }^{}\delta (C_{i}^{\alpha },C_{j}^{\alpha })\right).$$

Here, *γ* is a smoothing parameter, 〈ij〉 is the set of all the spots *j* that are neighbors of *i*, and *Z_i_* is the normalization factor. As suggested by (Zhao *et al.* (2021), we used *γ* = 2 for ST data, and *γ* = 3 for Visium data. The normalization factor *Z_i_* is calculated by aggregating the values of $p(C_{i}^{\alpha })$ for all possible cluster labels of the *i*-th spot. The Potts model encourages nearby spots to belong to the same cluster, thus assigning a higher likelihood to cluster assignments with better spatial continuity.

Lastly, we expect the spots belonging to the same cluster to have similar gene expressions (and thus similar PCs). Therefore, the correct cluster assignment should have a lower sum of variances of PCs within each cluster. Hence, we define the third quasi-likelihood component $q(y,C^{\alpha })$ as:


$$q(y,C^{\alpha })=\text{exp}\;\left(-\lambda \sum_{j=1}^{s}tr\left(\Sigma_{j}\right)\right),$$

where tr() denotes the trace of a matrix, and *λ* is a scaling factor that is set to 100 in this study. Therefore, the final goodness measure of the clustering output $v(y,C^{\alpha })$ is the product of the three components,


$$v(y,C^{\alpha })=q(y,C^{\alpha })p(y|C^{\alpha })p(C^{\alpha })=q(y,C^{\alpha })\prod_{i=1}^{n}p(y_{i}|C_{i}^{\alpha })p(C_{i}^{\alpha })$$

For AutoScribbleDom, we follow the exact same process, but in this case, we search the (*α*, *β*) hyperparameter space instead of just *α*.

### 2.8 Ground truth annotation procedure

In the case of the human DLPFC data, the ground truth annotations are done by Maynard *et al.* (2021) based on cytoarchitecture and selected gene markers. For the melanoma sample, the contour annotations over the histology image were done by Thrane *et al.* (2018); spot-level annotations did not exist. To generate spot-level annotations, we utilized the ST Spot Detector tool. Due to printing artifacts, the array used to create ST datasets may have positional variations. To address this issue, the tool provides corrected spot coordinates in both adjusted array coordinates and pixel coordinates on a high-resolution image. We then generated a mapping of array annotation based on the annotations made by Thrane *et al.* (2018) over the histology image.

In the process of manually annotating melanoma spots, we encountered two types of unannotated spots (Fig. 4c). Firstly, there were spots in the region that were not originally annotated by Thrane *et al.* (2018), the publishers of the dataset. Secondly, there were spots located at the borders of two regions, making it challenging to assign them to a specific region accurately. We treated both kinds of spots as unannotated while measuring clustering performances.

### 2.9 Performance measure

To objectively assess the similarity between cluster labels and manual annotation, which is regarded as the ground truth, we employed the ARI. Given a set *S* of *n* elements, and two groupings or partitions (e.g. clusterings) of these elements, namely $X=\{X_{1},X_{2},\ldots ,X_{r}\}$ and $Y=\{Y_{1},Y_{2},\ldots ,Y_{s}\}$, the overlap between X and Y can be summarized in a contingency table $\left[n_{ij}\right]$ where each entry *n_ij_* denotes the number of objects in common between *X_i_* and $Y_{j}:n_{ij}=|X_{i}\cap Y_{j}|$


$$\mathrm{ARI}=\frac{\sum_{ij}\left(\begin{array}{c} n_{ij}\\ 2 \end{array}\right)-\left[\sum_{i}\left(\begin{array}{c} a_{i}\\ 2 \end{array}\right)\sum_{j}\left(\begin{array}{c} b_{j}\\ 2 \end{array}\right)\right]/\left(\begin{array}{c} n\\ 2 \end{array}\right)}{\frac{1}{2}\left[\sum_{i}\left(\begin{array}{c} a_{i}\\ 2 \end{array}\right)+\sum_{j}\left(\begin{array}{c} b_{j}\\ 2 \end{array}\right)\right]-\left[\sum_{i}\left(\begin{array}{c} a_{i}\\ 2 \end{array}\right)\sum_{j}\left(\begin{array}{c} b_{j}\\ 2 \end{array}\right)\right]/\left(\begin{array}{c} n\\ 2 \end{array}\right)}$$

where $a_{i}=\sum_{j}n_{ij}$, $b_{j}=\sum_{i}n_{ij}$

## 3 Results

### 3.1 ScribbleDom outperforms state-of-the-art methods in 9 out of the 12 samples of the human DLPFC dataset

Table 1 shows the comparison of ScribbleDom with several state-of-the-art algorithms in terms of ARI. We chose to compare with BayesSpace, SC-MEB, SpaGCN, and Giotto as these are four very recently published spatial clustering algorithms. Additionally, SpaGCN integrates information from gene expression, spatial location, and histology for spatial domain identification. Since ScribbleDom also extracts some information from histology (through the form of expert scribbles), it made perfect sense to benchmark against SpaGCN. From the performance of the state-of-the-art spatial clustering methods, it is clear that spatial clustering algorithms perform much better than the non-spatial ones in spatial domain identification. Nevertheless, to be comprehensive, we wanted to benchmark ScribbleDom against a few non-spatial clustering algorithms. For this, we chose Gaussian mixture model (GMM) (Bishop 2006) and Louvain (De Meo *et al.* 2011). We have compared with BayesSpace, SpaGCN, and SC-MEB which are state-of-the-art methods. We have also compared with Giotto (Dries *et al.* 2021) and two non-spatial clustering algorithms, namely, GMM (Bishop 2006) and Louvain (De Meo *et al.* 2011). ScribbleDom equals or outperforms all the aforementioned state-of-the-art methods in 9 out of the 12 samples, improving the ARI by 51.90%, 47.40%, and 49.84% on average compared to BayesSpace, SC-MEB, and SpaGCN, respectively. Additionally, ScribbleDom equals or outperforms BayesSpace in 10, and SC-MEB in 11 samples. On the other hand, AutoScribbleDom equals or outperforms BayesSpace in 5 samples, and SC-MEB in 7 samples.

**Figure 3.**
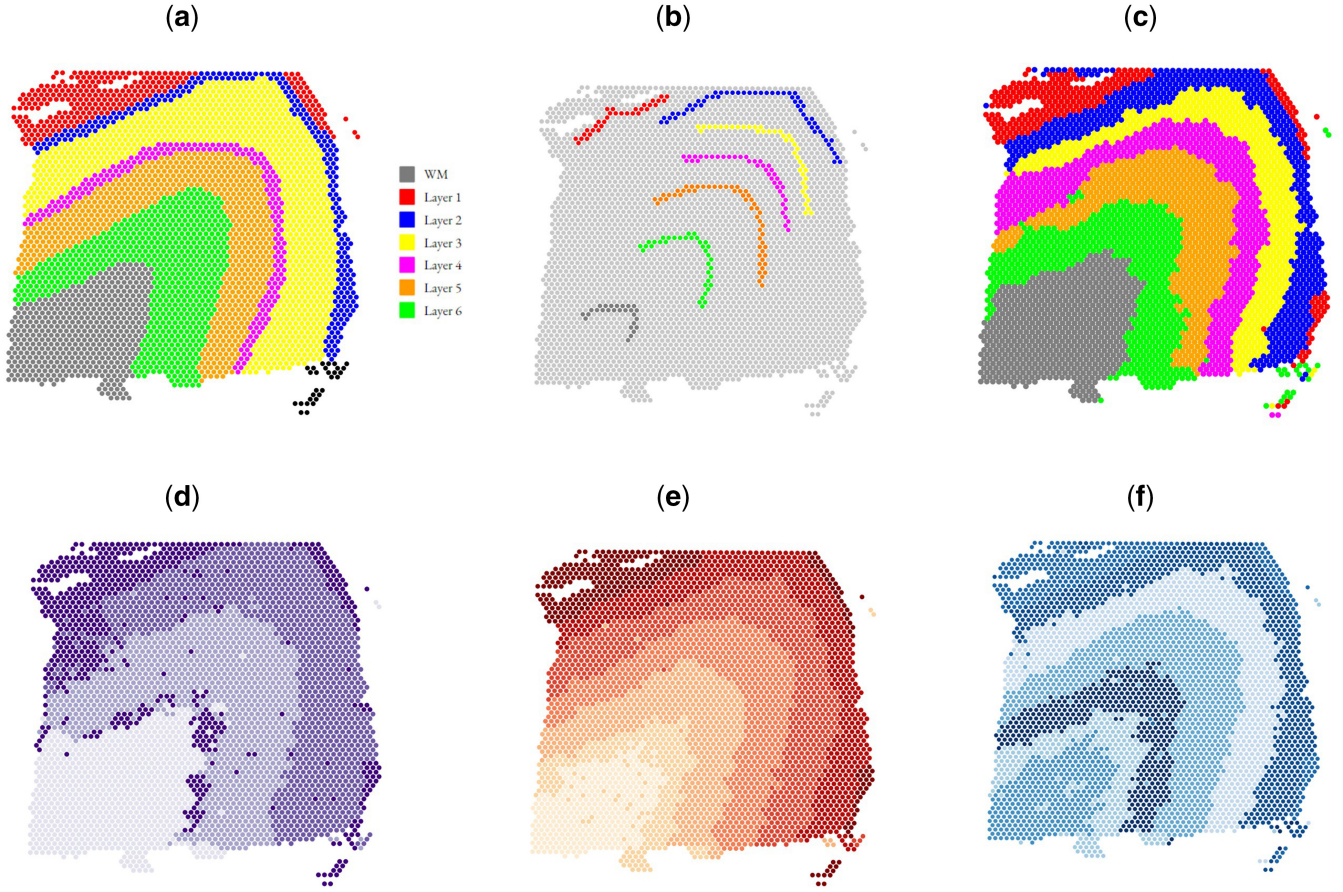
Comparison of various spatial domain detection algorithms on the DLPFC dataset, sample 151673. (a) Manual annotation (ground truth). (b) Scribbles by a human annotator. (c) Spatial domains detected by ScribbleDom. (d) Spatial domains detected by SC-MEB. (e) Spatial domains detected by BayesSpace. (f) Spatial domains detected by AutoScribbleDom.

These results imply significant quantitative superiority of ScribbleDom over state-of-the-art methods. Moreover, one of the main drawbacks of BayesSpace is the greater thickness of some layers compared to the manual annotation, e.g. layer 4 (colored magenta) of Fig. 3a. BayesSpace (Fig. 3f) predicted layer 4 to be much thicker than what the manual annotation suggests. ScribbleDom can identify layer 4 as a comparatively narrower layer (Fig. 3d) which better resembles the manual annotation.

### 3.2 ScribbleDom identifies narrow lymphoid tissue regions around the tumor in melanoma cancer sample

**Figure 4.**
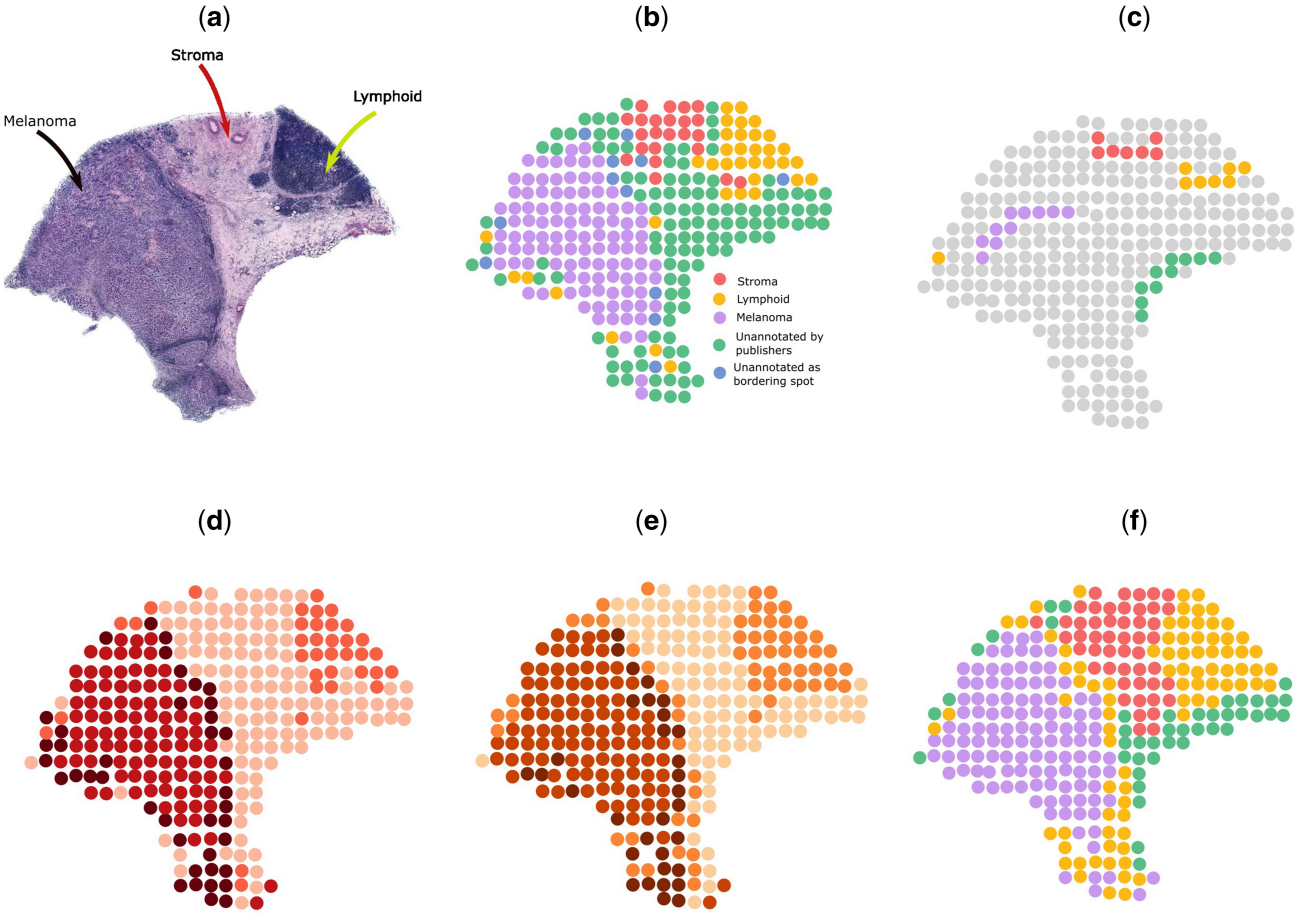
Analysis on melanoma ST sample. (a) Histology image. (b) Manual annotation. (c) Scribbles by a human annotator. (d) Spatial domains detected by BayesSpace. (e) Spatial domains detected by AutoScribbleDom. (f) Spatial domains detected by ScribbleDom.

We evaluated the performance of ScribbleDom on a melanoma sample from Thrane *et al.* (2018), and it outperforms BayesSpace by 15.54% in terms of ARI calculated on the manually annotated regions. The manual annotation (Fig. 4c) of the tissue revealed three distinct regions: melanoma, stroma, and lymphoid tissue, with an additional unannotated area. So, we conducted experiments with ScribbleDom as well as AutoScribbleDom using four clusters.

ScribbleDom captured the melanoma tumor region as a whole (Fig. 4g), in contrast to other methods such as BayesSpace (Fig. 4e), Giotto (Dries *et al.* 2021), and Louvain (De Meo *et al.* 2011), which tended to split the tumor into peripheral and central regions. Notably, our method also successfully identified the lymphoid tissues surrounding the tumor region, a feature shared only by Giotto, SC3 (Kiselev *et al.* 2017), and BayesSpace in subspot resolution. BayesSpace does not capture the lymphoid tissue bordering the tumor in its original resolution (Fig. 4e). On the other hand, AutoScribbleDom identified a periphery around the center of the tumor region, along with the surrounding lymphoid tissue (Fig. 4f).

We calculated the ARI (excluding the unannotated spots) to measure the agreement between spatial domains detected by ScribbleDom and the manual annotation. ScribbleDom achieved an ARI of 0.87 outperforming BayesSpace (0.75). On the other hand, AutoScribbleDom achieved an ARI of 0.74. Although slightly lower than the ARI achieved by BayesSpace, our method exhibited superior accuracy in capturing the presence of lymphoid tissues surrounding the tumor.

These findings highlight the efficacy of ScribbleDom in identifying narrow regions, such as lymphoid cells around the melanoma tumor in cancer tissue, demonstrating its superior performance compared to BayesSpace. Also, AutoScribbleDom autonomously identified most of the annotated lymphoid regions. These results underscore the potential of ScribbleDom and AutoScribbleDom in facilitating a better understanding of the tumor microenvironment.

### 3.3 ScribbleDom can differentiate tumor and non-tumor regions in human breast cancer tissue

**Figure 5.**
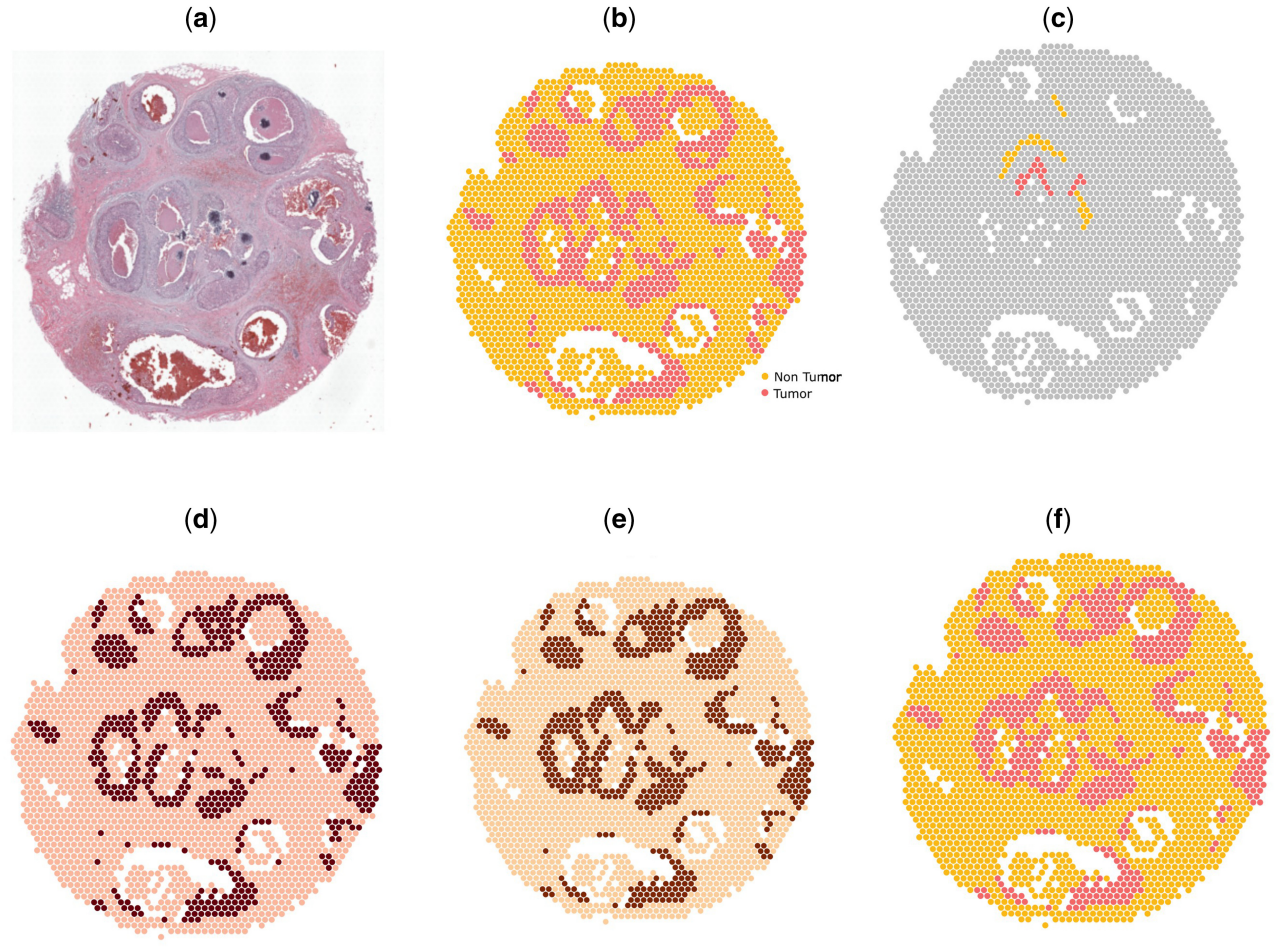
Analysis on human breast cancer dataset. (a) Histology image. (b) Manual annotation. (c) Scribbles by a human annotator. (d) Spatial domains detected by BayesSpace. (e) Spatial domains detected by AutoScribbleDom. (f) Spatial domains detected by ScribbleDom.

We applied ScribbleDom to analyze a human breast cancer Visium sample, and it performed at par with the results obtained using BayesSpace in both semi-supervised and unsupervised settings. The manual annotation of the sample consists of two distinct regions: tumor and non-tumor (Fig. 5c). With a limited amount of scribbles (Fig. 5d), ScribbleDom was able to successfully detect the tumors (Fig. 5g). ScribbleDom achieved an ARI of 0.82 (Fig. 5g). Even AutoScribbleDom obtained a competitive ARI of 0.83 (Fig. 5f). While this ARI is slightly lower than that of BayesSpace (0.84) (Fig. 5e), it is worth noting that ScribbleDom or AutoScribbleDom still effectively captured all the tumor regions in the human breast cancer sample.

## 4 Discussion

ScribbleDom detects spatial domains in ST tissue samples by leveraging gene expression, spatial neighborhood, and some prior knowledge about the sample. The prior information can either be from a human annotator for some of the spots in the form of scribbles on histology image, or generated from an unsupervised non-spatial clustering algorithm (e.g. mclust). We named the later approach AutoScribbleDom. The output generated by ScribbleDom provides valuable insights into the transcriptionally homogeneous tissue regions within the spatial transcriptomics data. In comparison to existing state-of-the-art methods, ScribbleDom demonstrates superior accuracy in detecting spatial domains whereas AutoScribbleDom too shows competitive results.

Many recent methods incorporate histology information for spatial domain identification. However, not all spatial transcriptomics technology can generate RNA data and histology in a matching way. This may decrease the quality of histology to spot mapping. ScribbleDom can overcome this limitation as it does not require a one-to-one mapping from histology to spots. In the case where one-to-one histology to spot mapping is not present, the scribbled spots can potentially be derived from scribbles over the histology by an estimation method. So, scribbling over the spots of ST data is a potential future work.

Table 1 shows the superiority of ScribbleDom quantitatively, and Fig. 3g shows the qualitative superiority of ScribbleDom for sample 151673 of the human DLPFC dataset, where the thickness of the layers is better predicted by ScribbleDom (see for example, the thickness of layer 4, colored magenta in manual annotation). Figures for other samples can be found in the Supplementary data. Moreover, in the analysis of the melanoma and human breast cancer datasets, ScribbleDom successfully detected the tumor regions. Specifically, in the case of melanoma, we were able to capture the lymphoid tissues that border the melanoma region using both the semi-supervised (Fig. 4g) and unsupervised (Fig. 4f) approaches. Furthermore, ScribbleDom outperformed BayesSpace in terms of ARI, demonstrating a more accurate identification of the melanoma and lymphoid tissue regions. ScribbleDom’s capability to identify different regions of the tumor neighborhood suggests its potential use in detecting narrow regions such as lymphoid tissues. The ability to segment and identify specific regions within a tumor can aid in understanding the composition and characteristics of the tumor neighborhood.

Our experiments have demonstrated that ScribbleDom achieves high accuracy in detecting spatial domains within tissue regions when expert scribbles are available. This highlights ScribbleDom’s ability to leverage domain knowledge provided by expert annotators to identify different tissue domains. We have introduced a hyperparameter, *α*, to control the relative importance of scribbles and gene expression similarity across spots. Additionally, ScribbleDom incorporates inception blocks to capture multi-scale features within the tissue region, enabling accurate detection of various tissue domains, such as smooth layers in human DLPFC samples, narrow bordering regions of lymphoid tissues around tumor regions in melanoma, and scattered tumors across different regions in human breast cancer dataset. AutoScribbleDom also shows competitive results compared to state-of-the-art methods. It utilizes a non-spatial clustering algorithm (mclust) to generate automated scribbles and uses them for the identification of spatial domains. However, the automated scribbles generated by mclust may not accurately represent the spatial domain, due to its (mclust) inability to incorporate spatial information. To mitigate potential inaccuracies, we randomly drop some scribbled spots in each iteration, controlling the dropout rate with another parameter *β*. This approach helps AutoScribbleDom generalize the information from similar spots and reduces the impact of inaccurate automated scribbles.

Additionally, instead of fixing the hyperparameters at specific values, we performed a grid search across a range of hyperparameter values. We selected the hyperparameter values that maximize an ARI independent goodness score. The goodness is determined from the clustering likelihood, the conditional likelihood of the gene expression of spots given the clustering and a penalty term that tends to reduce variance of the PCs within the same cluster. Our proposed goodness score can potentially be used to compare clustering output of various algorithms and can help practitioners pick the best one.

Although there has not been a thorough benchmarking on the existing ST domain detection methods, the publications suggest that no method is an indisputable winner in every dataset. This leaves practitioners like clinicians and experimental biologists a bit helpless—on one hand, it is difficult to fine-tune the model parameters to obtain the domains that conform to known biology; on the other hand, the existing methods cannot directly incorporate any form of human supervision. Thus, ScribbleDom fills in a unique niche: (i) it is a versatile method that works satisfactorily both with and without human supervision and (ii) it can work with minimal human supervision, in the form of scribbles. ScribbleDom thus broadens the options for practitioners, especially for domain detection in complex tissues, e.g. from advanced disease states. Another important application of ScribbleDom can be in helping practitioners and method developers efficiently create ground truth annotations without inducing bias, e.g. in demarcating the precise domain boundaries.

ScribbleDom is generalizable to other sequencing-based technologies as well [e.g. Stereo-seq (Wei *et al.* 2022)]. These technologies capture cells in spots (beads) arranged in grids, and ScribbleDom’s convolutions (its basic blocks) work seamlessly on any grid-structured data. With minor preprocessing, ScribbleDom is also applicable to fluorescent in situ hybridization (FISH) based techniques. In these cases, ScribbleDom will need a preprocessed input where one has overlaid a grid structure on the spatial transcriptomic measurements. Alternately, graph convolutions can be implemented for FISH-based data, which is left as a future work.

ScribbleDom is extensible and can be pretrained if more datasets are available in the future. It enables the human-in-the-loop paradigm in the process of machine learning for the identification of spatial domains in the realm of spatial transcriptomics by incorporating prior annotations obtained from human experts. This means that a clinician is not limited to a fixed output provided by an unsupervised method. Instead, by inputting different scribbles, they can obtain diverse outputs, making the spatial domain identification process more robust and adaptable.

## 5 Conclusion

One of the “eleven grand challenges in single-cell data science” (Lahnemann *et al.* 2020) is to find patterns in spatially resolved measurements. We have incorporated a semi-supervised deep learning pipeline in this work to address that challenge. Inspired by similarities in the problem in hand to the image segmentation problem in the computer vision community, we started our endeavors from a state-of-the-art pipeline to solve the later problem. We then made considerable changes to the model to adapt it to spatial transcriptomics data. Furthermore, we made important changes in the model architecture to capture spatial domain features at different resolutions. Our method, ScribbleDom, allows for the incorporation of expert knowledge in the form of scribbles. The method can also function in a fully unsupervised manner when input from an expert is not available. Through a benchmark study with state-of-the-art methods on the human DLPFC, melanoma, and human breast cancer datasets, we have demonstrated the superiority of our semi-supervised approach. In the future, we can pretrain our model as more manually annotated data become available. The ScribbleDom package is well documented and easy to use. We hope that biologists will use ScribbleDom to identify spatial domains in ST samples that would benefit them for downstream analyses which will lead to potential discoveries in the field of bioinformatics.

## Supplementary Material

btad594_Supplementary_Data

## Data Availability

Human Dorsolateral prefrontal cortex (DLPFC) raw spatial data for all the 12 samples is publicly available in spatialLIBD website (https://research.libd.org/spatialLIBD/) under the “raw data” header. Human breast cancer: ductal carcinoma in situ, invasive carcinoma raw count matrix and tissue spatial positions are publicly available in 10xgenomics website (https://www.10xgenomics.com/resources/datasets/human-breast-cancer-ductal-carcinoma-in-situ-invasive-carcinoma-ffpe-1-standard-1-3-0) under the “Output Files” header. Melanoma raw count matrix can be downloaded from Spatial Research website (https://www.spatialresearch.org/wp-content/uploads/2019/03/ST-MelanomaDatasets_1.zip); row/column coordinates are obtained from column labels of the raw count matrix.
