# Supplementary material for: ScribbleDom: using scribble-annotated histology images to identify domains in spatial transcriptomics data
Source: Bioinformatics. 2023 Sep 26;39(10):btad594. doi: 10.1093/bioinformatics/btad594 (PMC10564617; doi:10.1093/bioinformatics/btad594)
Supplement: btad594_Supplementary_Data [file btad594_supplementary_data.pdf]

Supplementary file to ScribbleDom: Using Scribble-Annotated Histology Images to Identify Domains in Spatial Transcriptomics Data

Mohammad Nuwaisir Rahman[1], Abdullah Al Noman[1], Abir Mohammad Turza[1], Mohammed Abid Abrar[2], Md. Abul Hassan Samee[3,*], and M. Saifur Rahman[1,*]

[1]Bangladesh University of Engineering and Technology, Department of Computer Science and Engineering, Dhaka, 1000, Bangladesh
[2]Brac University, Department of Computer Science and Engineering, Dhaka, 1212, Bangladesh
[3]Baylor College of Medicine, Department of Integrative Physiology, Houston, Texas, 77030, United States of America
[*]corresponding author(s): Md. Abul Hassan Samee (samee@bcm.edu) and M. Saifur Rahman (mrahman@cse.buet.ac.bd)

ABSTRACT

In this supplementary file, we provide more details on the hyperparameter tuning. The spatial domain identified for human breast cancer, melanoma and only sample 151673 of the human DLPFC dataset was showcased in the main text. Here, we provide the heatmaps for all the other human DLPFC samples. Also, we provide detailed results after a grid search to find optimal hyperparameters.

Choosing number of top principal components for the human breast cancer sample

We have chosen the top 15 principal components from the 50 top principal components for the human breast cancer sample. The scree plot (Figure 1) demonstrates that 15 principal components would capture a significant amount of the dataset's variability.

Hyperparameter Tuning

As described in the main text, ScribbleDom selects optimal hyperparameter α (pair of parameters (α,β) in case of AutoScribbleDom) using a goodness measure. In our experiments conducted on the human DLPFC dataset (sample 151670) using expert annotations in the form of scribbles, we observed a strong correlation between this goodness measure and the Adjusted Rand Index (ARI).

Figure 2 shows the significance of incorporating the variance component (the third component) into this scoring function, denoted as $q(y,C^{\alpha})$. As depicted in Figure 2(a), when the scoring function does not include this component, a weak correlation ($\rho = -0.046$) is observed between the score and the corresponding ARI. However, when the variance component is included, a stronger correlation ($\rho = 0.642$) is observed (Figure 2(b)). This finding highlights the importance of considering the variance component in the optimization criteria. Figures 2(d) and 2(e) display the spatial clustering with the best scores when the scoring function does not and does include the variance component respectively. Both outputs are presented alongside the manual annotation shown in Figure 2(c).

Hyperparamter selected after grid search

The optimal hyperparameter values selected by ScribbleDom after grid search are shown for human DLPFC (Table 1), melanoma (Table 2) and human breast cancer (Table 3) samples. For grid search we have iterated over the value of α over 0.05 to 0.95 and β over 0.25 to 0.4 with an interval of 0.05.

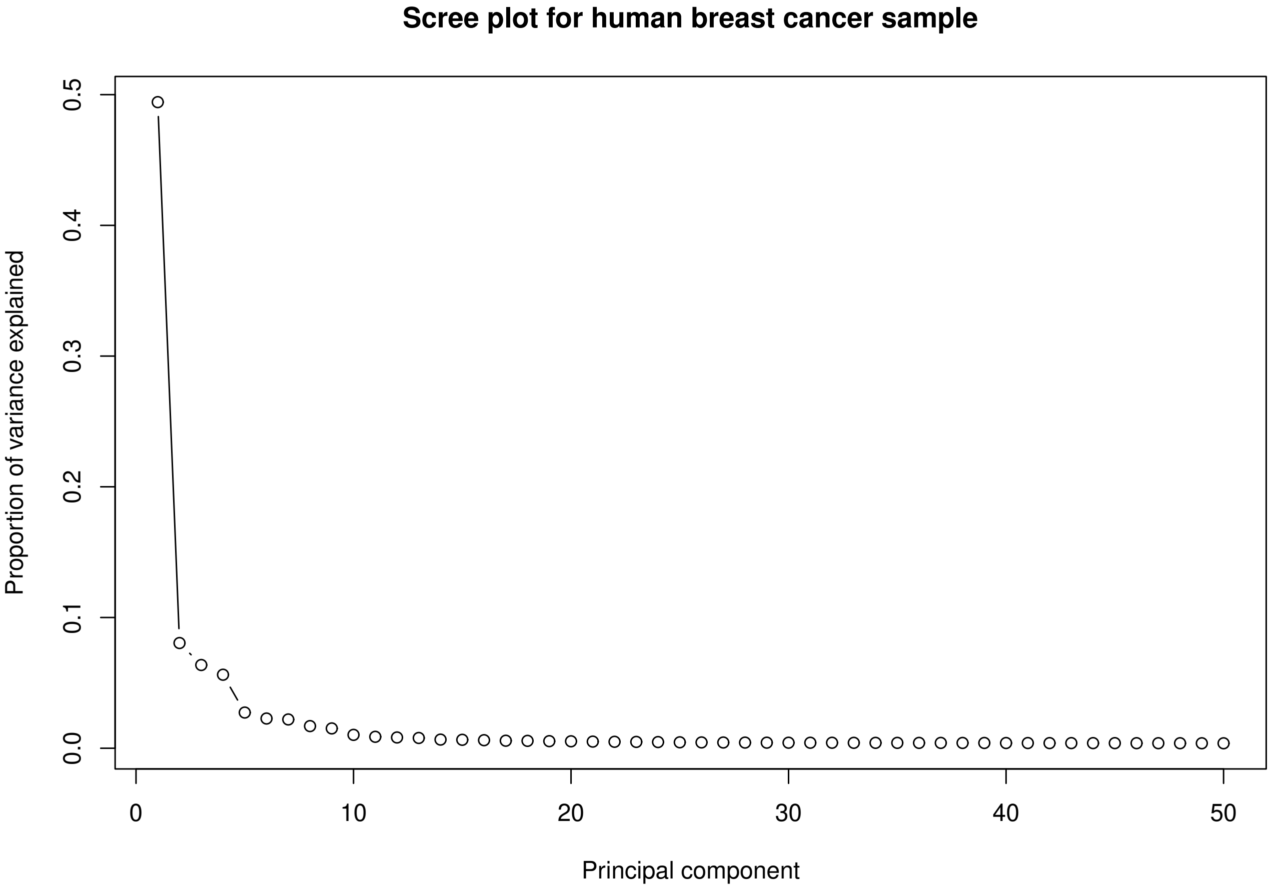

Figure 1. The scree plot for the human breast cancer sample.

Table 1. Hyperparamter selected for human DLPFC samples.

Sample	ScribbleDom (scribble initialization)	AutoScribbleDom (mclust initialization)	
	α	α	β
151507	0.85	0.90	0.40
151508	0.75	0.25	0.30
151509	0.90	0.90	0.35
151510	0.80	0.95	0.25
151669	0.95	0.85	0.30
151670	0.50	0.90	0.25
151671	0.95	0.85	0.40
151672	0.95	0.70	0.35
151673	0.90	0.90	0.40
151674	0.75	0.15	0.40
151675	0.95	0.75	0.30
151676	0.95	0.90	0.25

Spatial domains in the Human DLPFC Dataset

The spatial domains detected for the rest of the samples of Human DLPFC dataset, as obtained from ScribbleDom (both expert scribble scheme and mclust generated automated scribble scheme), BayesSpace[1], and SC-MEB[2], along with manual

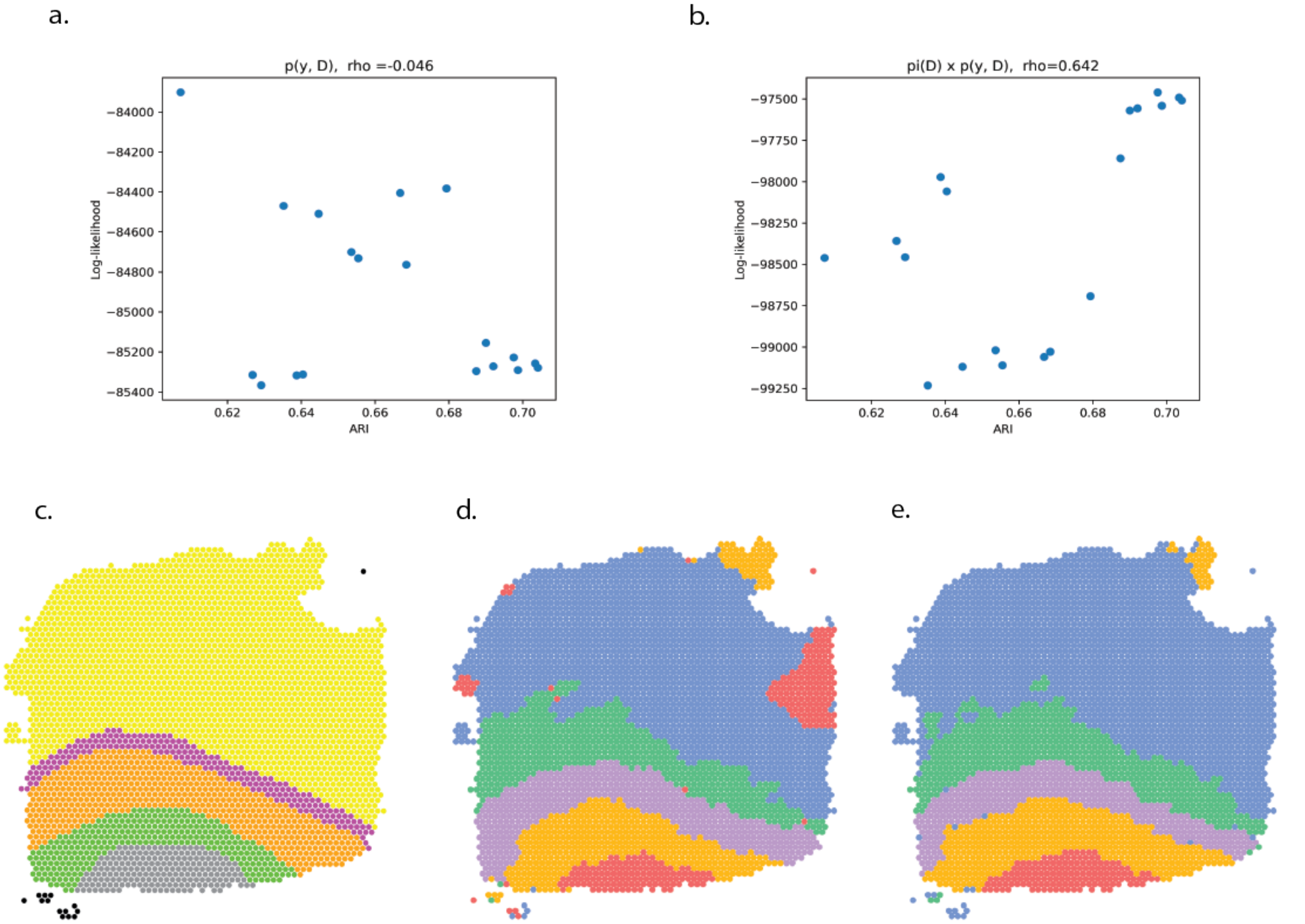

Figure 2. Importance of using variance component with joint likelihood, shown in sample 151670 of the human DLPFC dataset. a. Correlation between likelihood and ARI with optimization scheme, solely using joint-likelihood ($q(y, C^{\alpha})$). **b.** Correlation between likelihood and ARI with optimization scheme, when variance component is used with joint-likelihood ($q(y, C^{\alpha})$). **c.** Manual annotation (ground truth). **d.** Spatial domains detected with optimzation scheme using joint likelihood. **e.** Spatial domains detected with optimization scheme, when variance component is used with joint likelihood.

Table 2. Hyperparameter selected for melanoma sample.

Sample	ScribbleDom (scribble initialization)	ScribbleDom (mclust initialization)	
	α	α	β
ST_mel1_rep2	0.95	0.70	0.40

Table 3. Hyperparameter selected for breast cancer sample.

Sample	ScribbleDom (scribble initialization)	ScribbleDom (mclust initialization)	
	α	α	β
Human breast cancer	0.25	0.40	0.35

annotation and human annotator's scribbles are shown in this section. The result is generated after a grid search to find optimal hyperparameters. To generate the sc-meb outputs, a seed value of 121 was utilized.

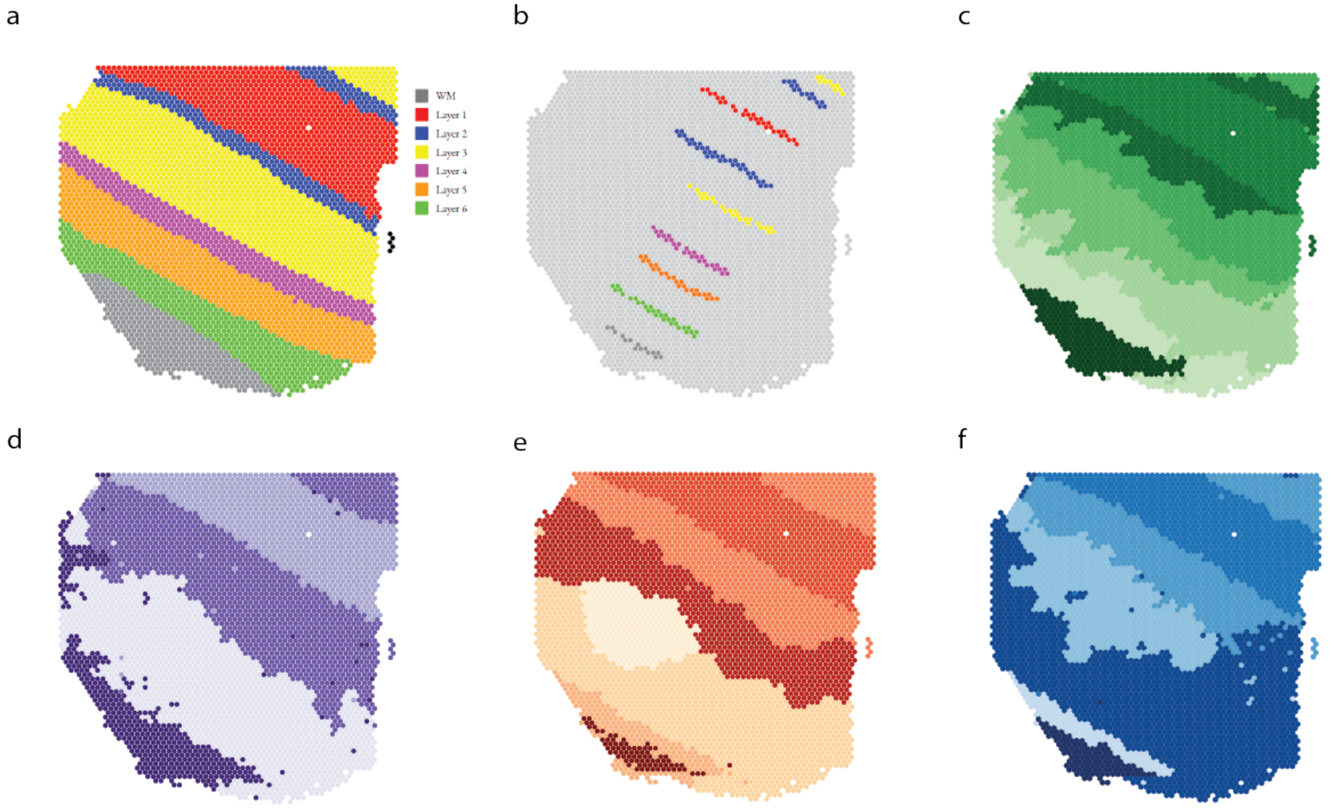

Figure 3. Comparison of various spatial domain detection algorithms on the DLPFC dataset, sample 151507. **a.** Manual annotation (ground truth). **b.** Scribbles by an annotator. **c.** Spatial domains detected by ScribbleDom, based on annotator's scribbles. **d.** Spatial domains detected by SC-MEB. **e.** Spatial domains detected by BayesSpace. **f.** Spatial domains detected by AutoScribbleDom.

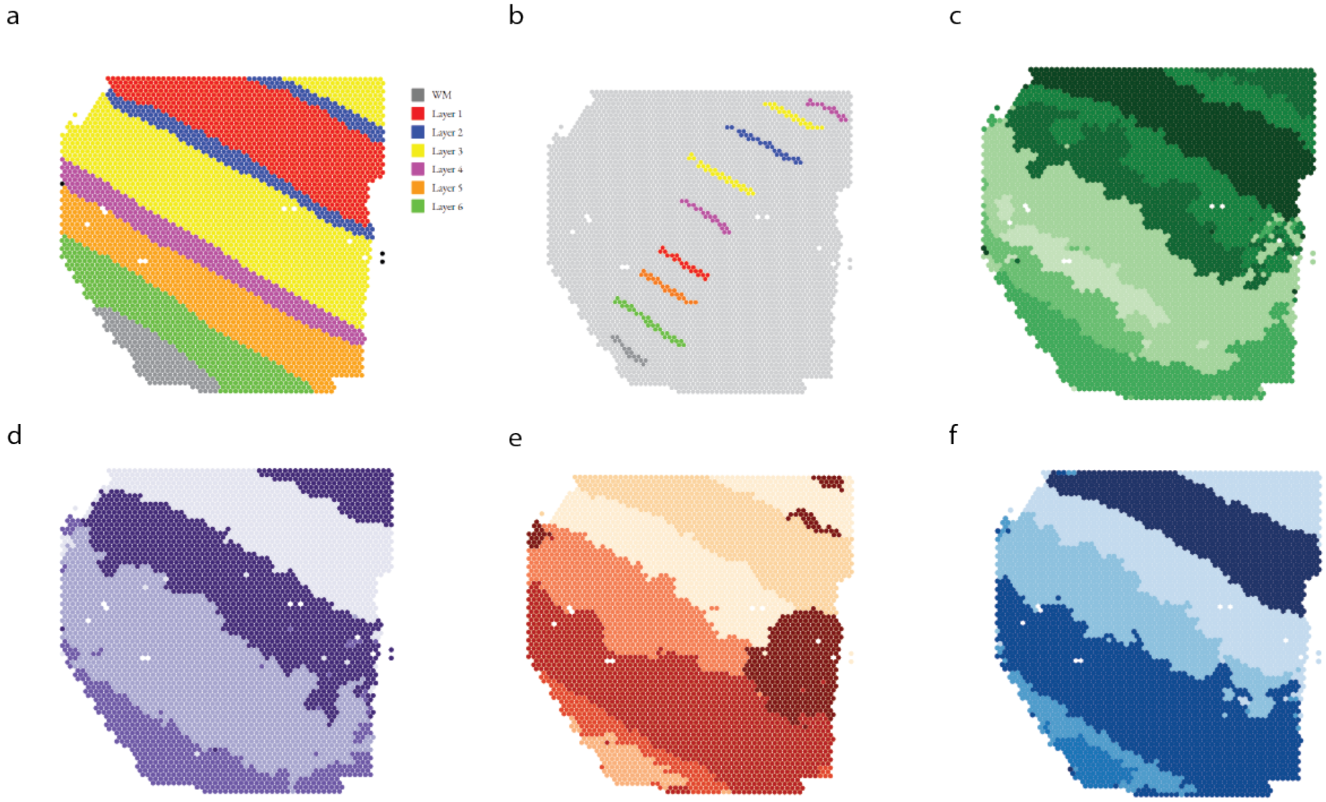

Figure 4. Comparison of various spatial domain detection algorithms on the DLPFC dataset, sample 151508. **a.** Manual annotation (ground truth). **b.** Scribbles by an annotator. **c.** Spatial domains detected by ScribbleDom, based on annotator's scribbles. **d.** Spatial domains detected by SC-MEB. **e.** Spatial domains detected by BayesSpace. **f.** Spatial domains detected by AutoScribbleDom.

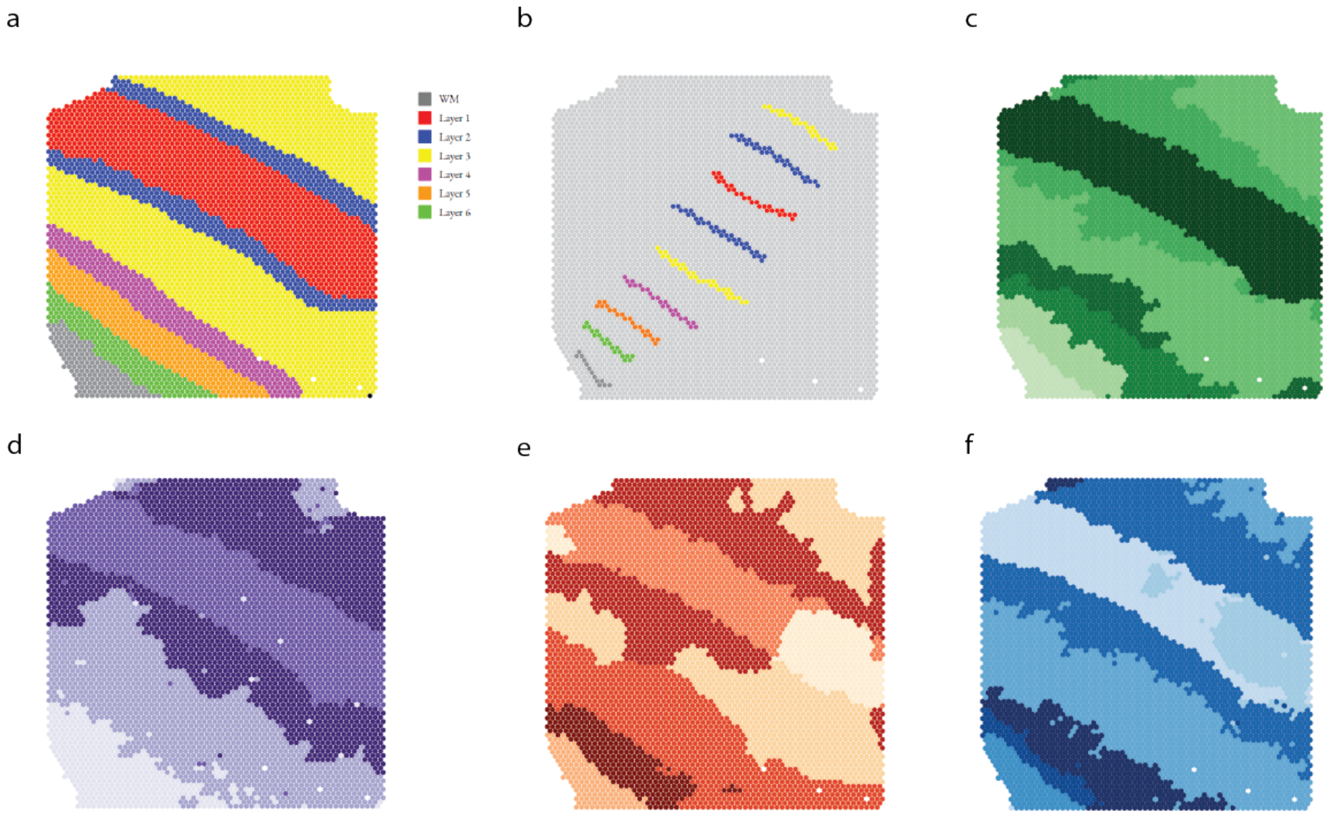

Figure 5. Comparison of various spatial domain detection algorithms on the DLPFC dataset, sample 151509. **a.** Manual annotation (ground truth). **b.** Scribbles by an annotator. **c.** Spatial domains detected by ScribbleDom, based on annotator's scribbles. **d.** Spatial domains detected by SC-MEB. **e.** Spatial domains detected by BayesSpace. **f.** Spatial domains detected by AutoScribbleDom.

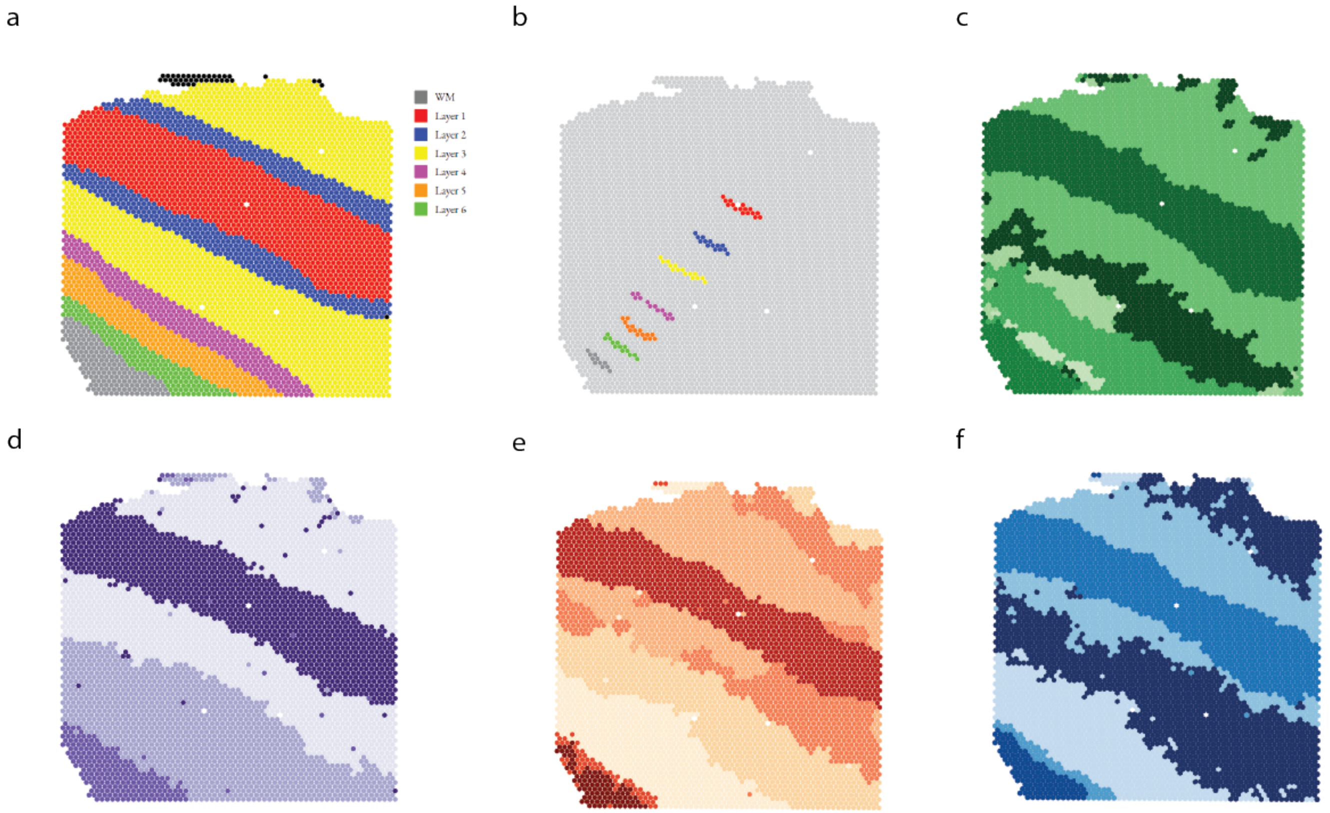

Figure 6. Comparison of various spatial domain detection algorithms on the DLPFC dataset, sample 151510. **a.** Manual annotation (ground truth). **b.** Scribbles by an annotator. **c.** Spatial domains detected by ScribbleDom, based on annotator's scribbles. **d.** Spatial domains detected by SC-MEB. **e.** Spatial domains detected by BayesSpace. **f.** Spatial domains detected by AutoScribbleDom.

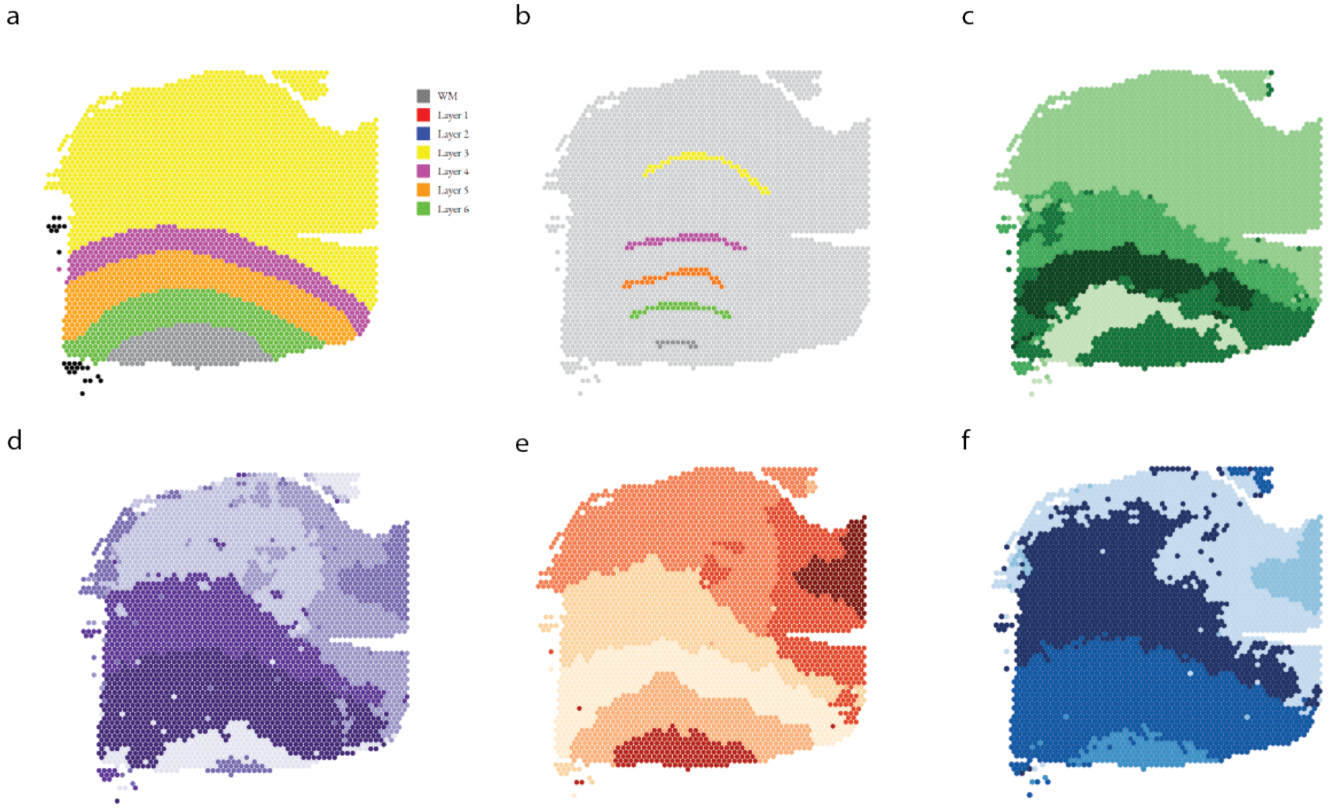

Figure 7. Comparison of various spatial domain detection algorithms on the DLPFC dataset, sample 151669. **a.** Manual annotation (ground truth). **b.** Scribbles by an annotator. **c.** Spatial domains detected by ScribbleDom, based on annotator's scribbles. **d.** Spatial domains detected by SC-MEB. **e.** Spatial domains detected by BayesSpace. **f.** Spatial domains detected by AutoScribbleDom.

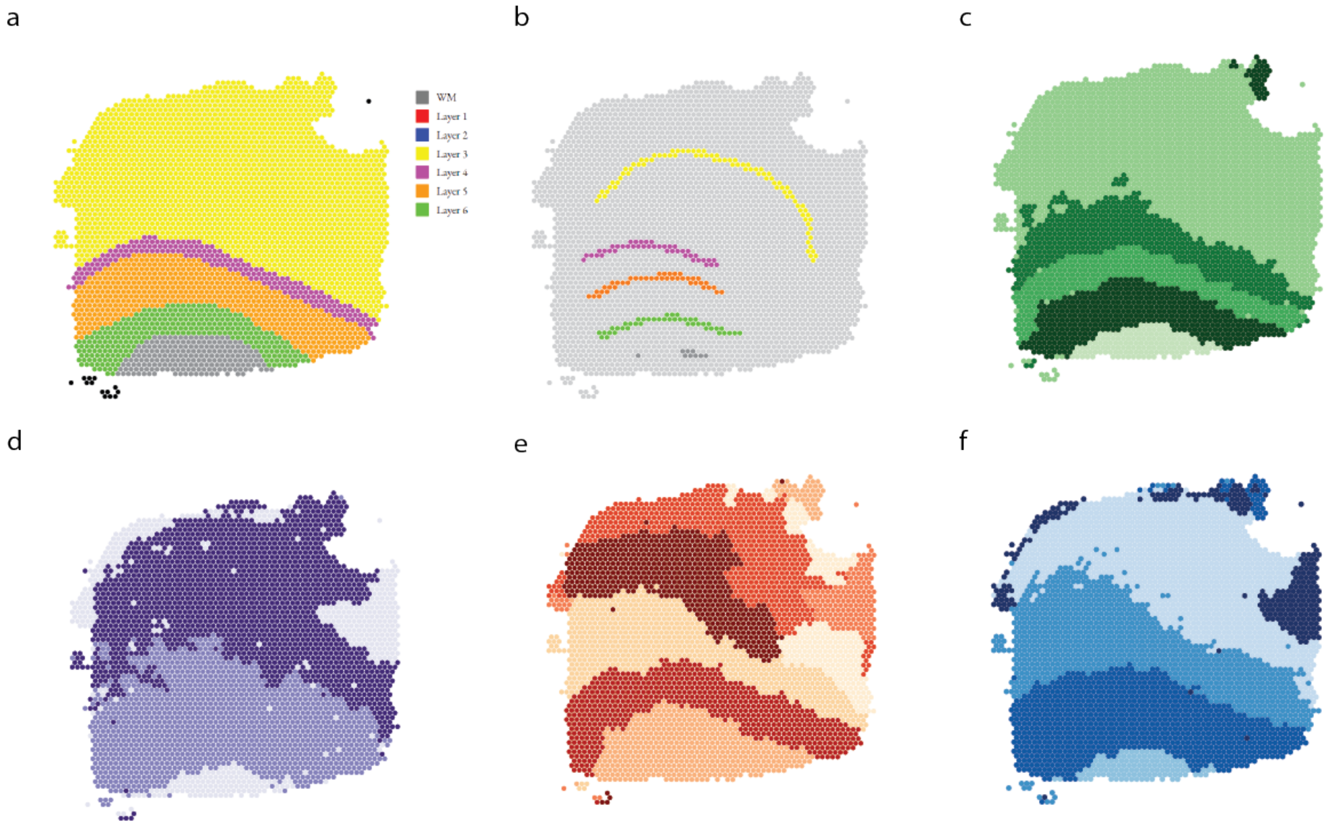

Figure 8. Comparison of various spatial domain detection algorithms on the DLPFC dataset, sample 151670. **a.** Manual annotation (ground truth). **b.** Scribbles by an annotator. **c.** Spatial domains detected by ScribbleDom, based on annotator's scribbles. **d.** Spatial domains detected by SC-MEB. **e.** Spatial domains detected by BayesSpace. **f.** Spatial domains detected by AutoScribbleDom.

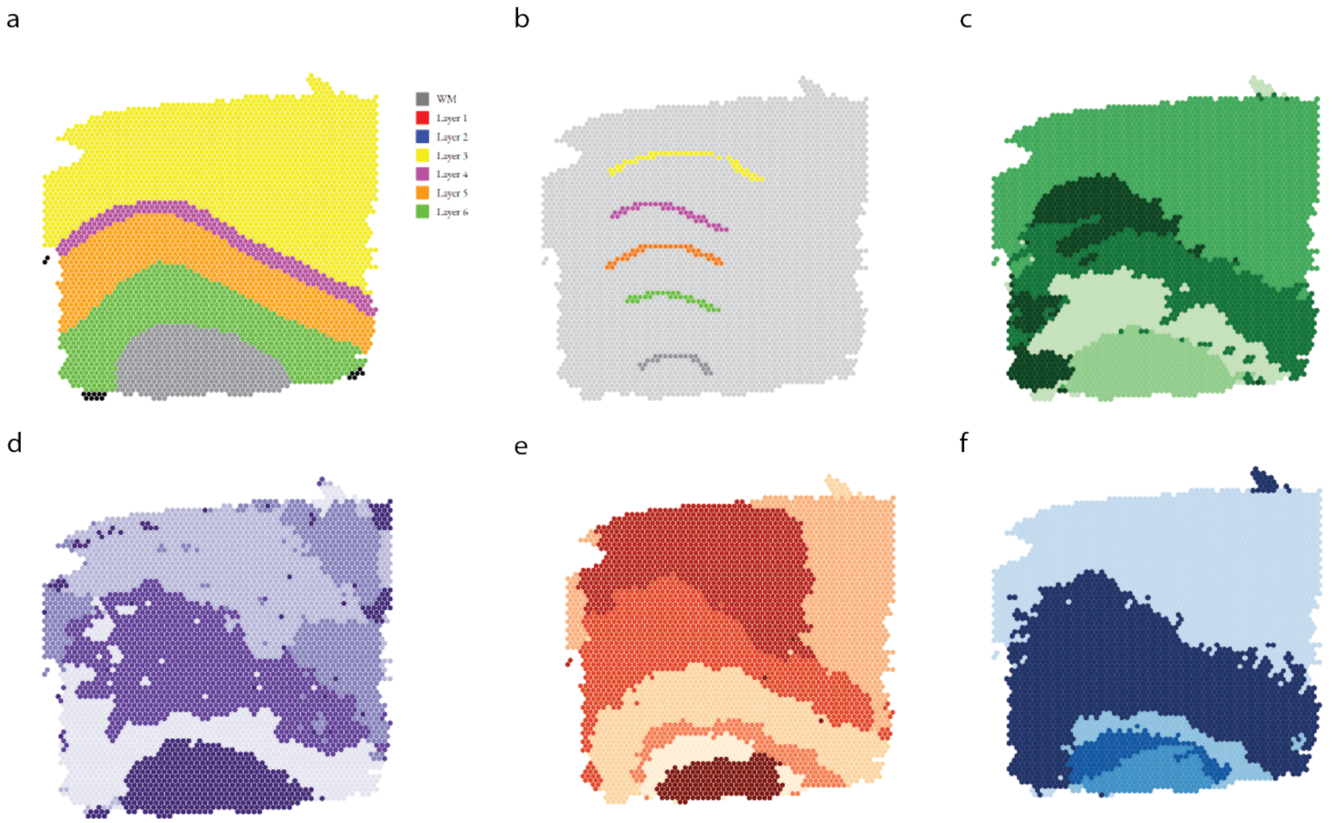

Figure 9. Comparison of various spatial domain detection algorithms on the DLPFC dataset, sample 151671. **a.** Manual annotation (ground truth). **b.** Scribbles by an annotator. **c.** Spatial domains detected by ScribbleDom, based on annotator's scribbles. **d.** Spatial domains detected by SC-MEB. **e.** Spatial domains detected by BayesSpace. **f.** Spatial domains detected by AutoScribbleDom.

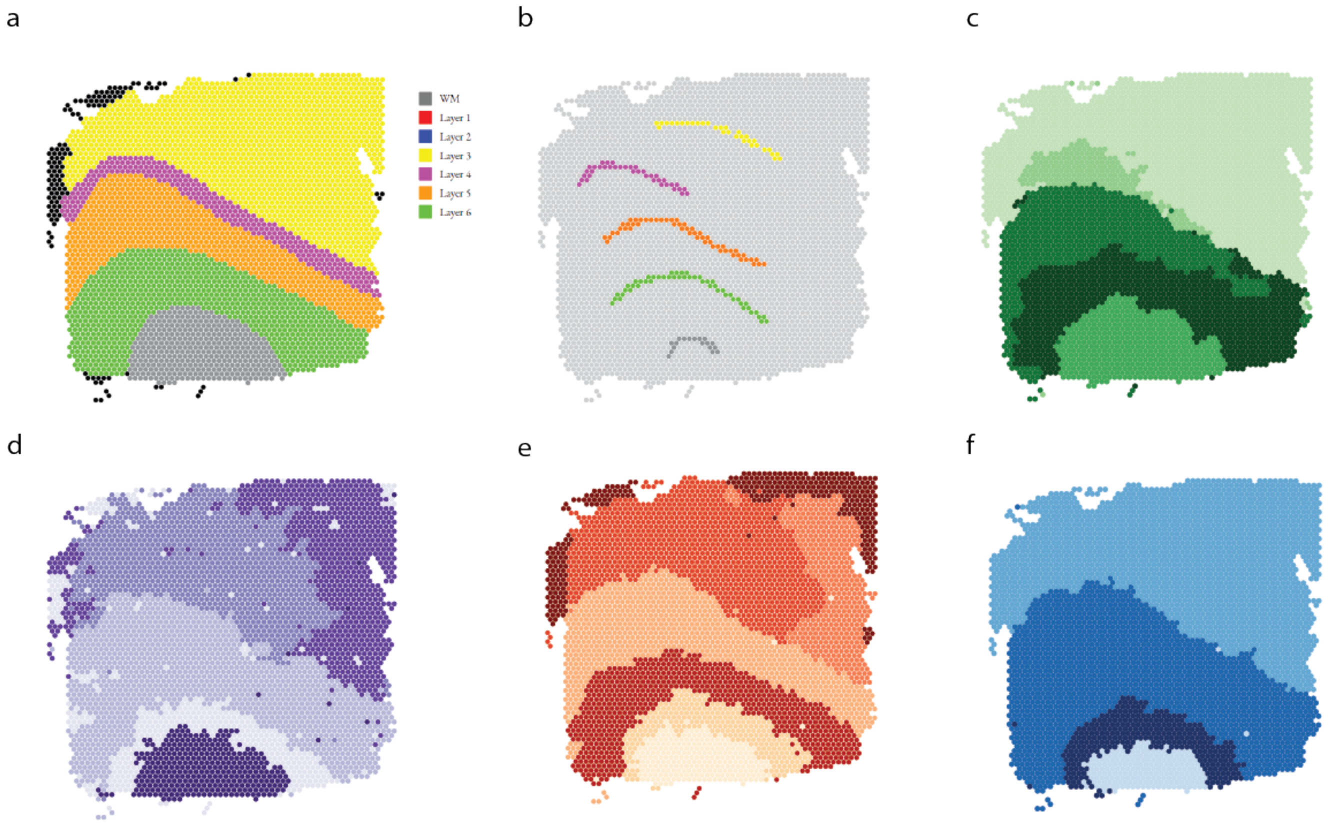

Figure 10. Comparison of various spatial domain detection algorithms on the DLPFC dataset, sample 151672. **a.** Manual annotation (ground truth). **b.** Scribbles by an annotator. **c.** Spatial domains detected by ScribbleDom, based on annotator's scribbles. **d.** Spatial domains detected by SC-MEB. **e.** Spatial domains detected by BayesSpace. **f.** Spatial domains detected by AutoScribbleDom.

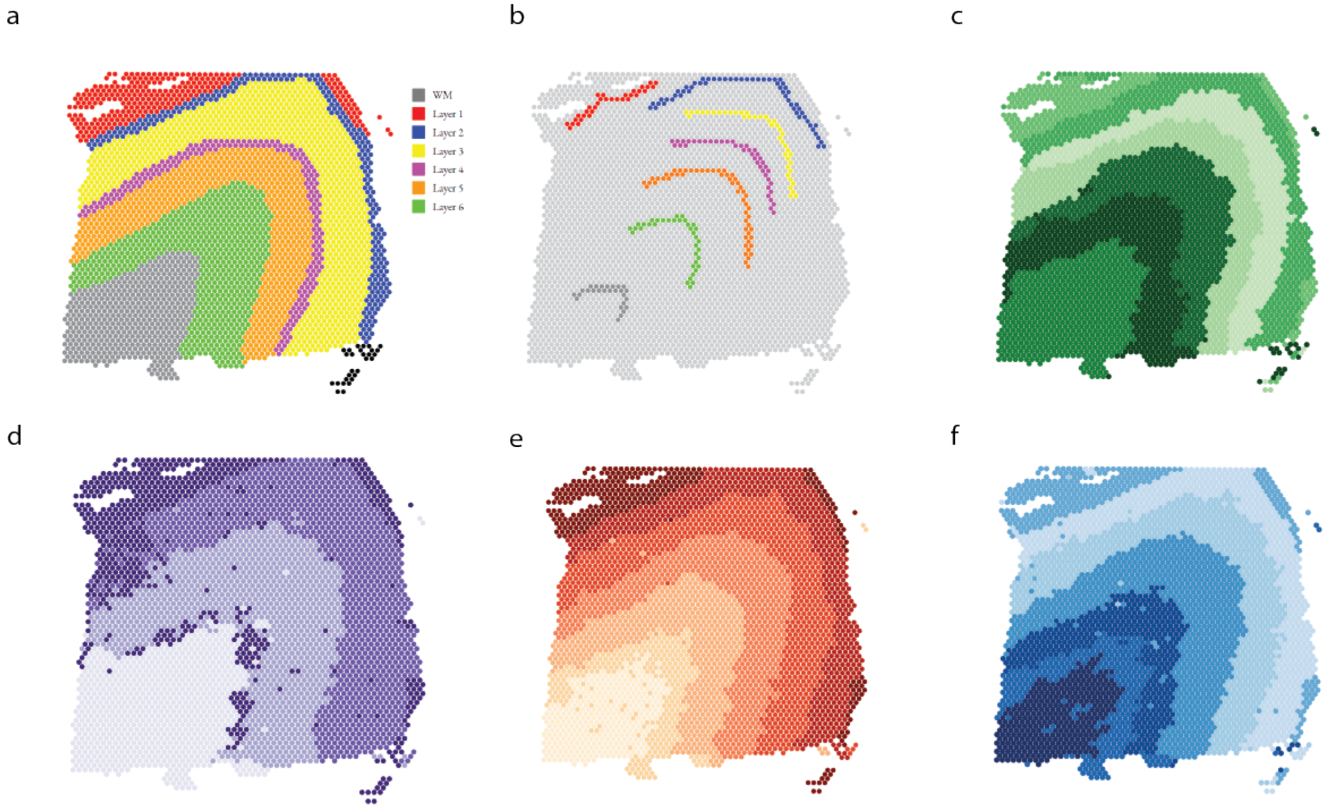

Figure 11. Comparison of various spatial domain detection algorithms on the DLPFC dataset, sample 151673. **a.** Manual annotation (ground truth). **b.** Scribbles by an annotator. **c.** Spatial domains detected by ScribbleDom, based on annotator's scribbles. **d.** Spatial domains detected by SC-MEB. **e.** Spatial domains detected by BayesSpace. **f.** Spatial domains detected by AutoScribbleDom.

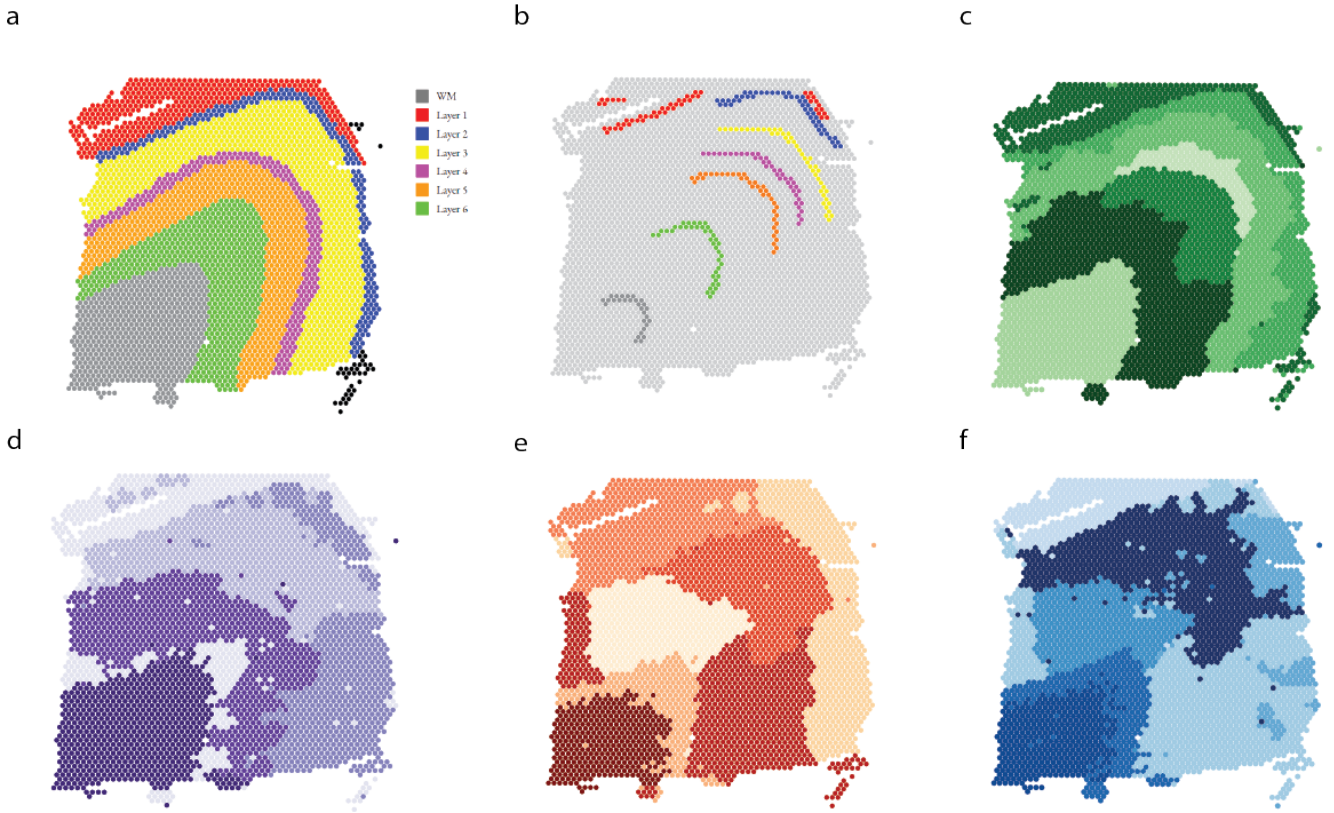

Figure 12. Comparison of various spatial domain detection algorithms on the DLPFC dataset, sample 151674. **a.** Manual annotation (ground truth). **b.** Scribbles by an annotator. **c.** Spatial domains detected by ScribbleDom, based on annotator's scribbles. **d.** Spatial domains detected by SC-MEB. **e.** Spatial domains detected by BayesSpace. **f.** Spatial domains detected by AutoScribbleDom.

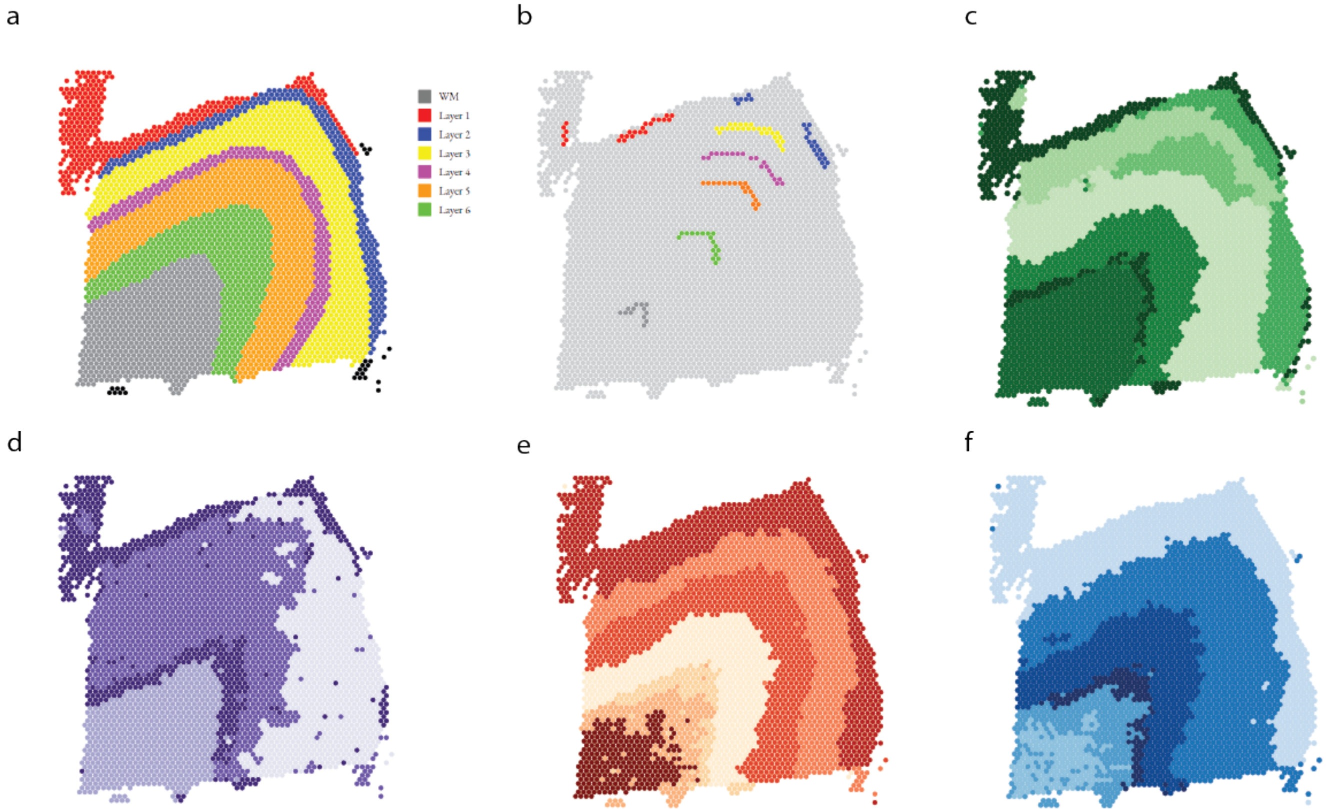

Figure 13. Comparison of various spatial domain detection algorithms on the DLPFC dataset, sample 151675. a. Manual annotation (ground truth). **b.** Scribbles by an annotator. **c.** Spatial domains detected by ScribbleDom, based on annotator's scribbles. **d.** Spatial domains detected by SC-MEB. **e.** Spatial domains detected by BayesSpace. **f.** Spatial domains detected by AutoScribbleDom.

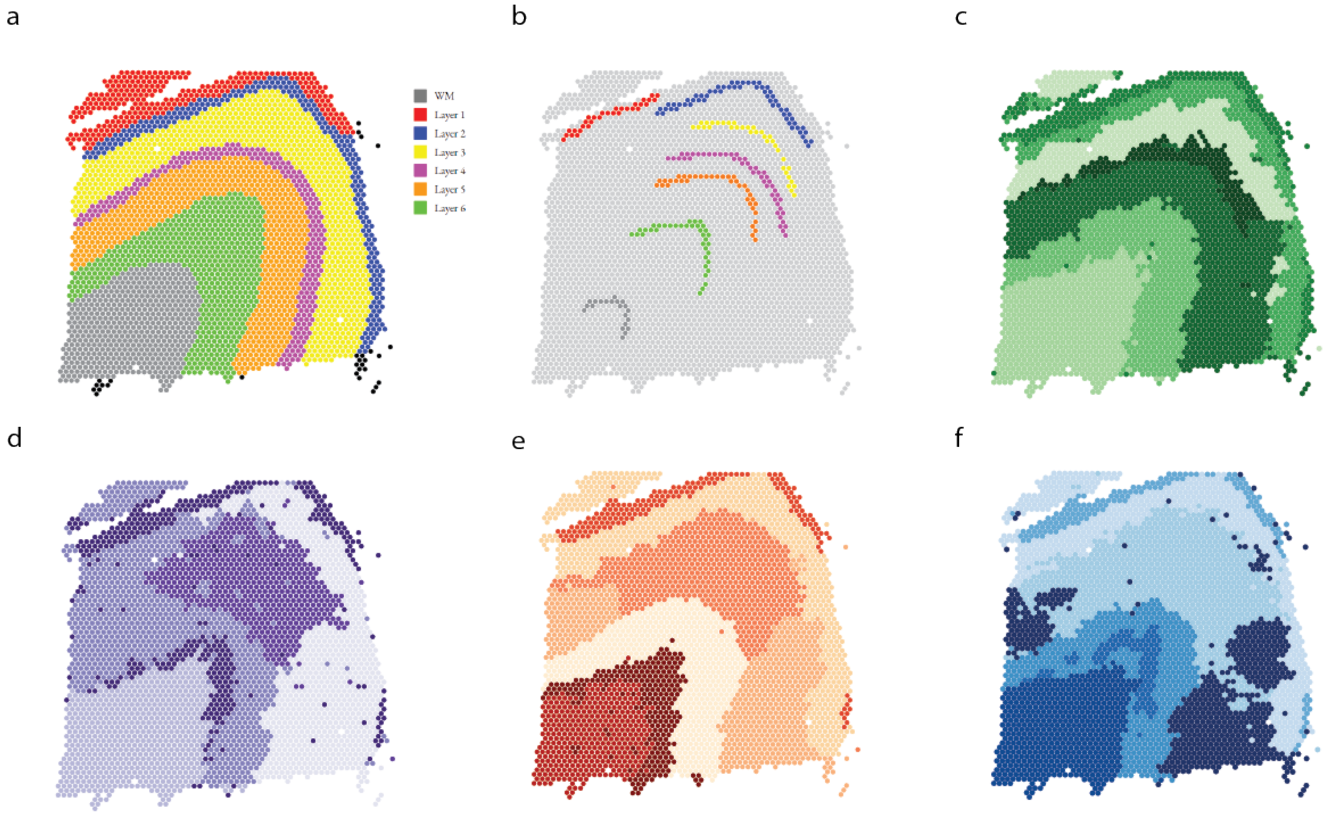

Figure 14. Comparison of various spatial domain detection algorithms on the DLPFC dataset, sample 151676. **a.** Manual annotation (ground truth). **b.** Scribbles by an annotator. **c.** Spatial domains detected by ScribbleDom, based on annotator's scribbles. **d.** Spatial domains detected by SC-MEB. **e.** Spatial domains detected by BayesSpace. **f.** Spatial domains detected by AutoScribbleDom.

References

1. Zhao, E. *et al.* Spatial transcriptomics at subspot resolution with BayesSpace. *Nat Biotechnol* **39**, 1375–1384 (2021).

2. Yang, Y. *et al.* SC-MEB: spatial clustering with hidden Markov random field using empirical Bayes. *Briefings Bioinforma.* **23**, 10.1093/bib/bbab466 (2021). Bbab466, https://academic.oup.com/bib/article-pdf/23/1/bbab466/42230744/bbab466.pdf.
